# Combined Effects of Sodium D-Isoascorbate, Phospholipids and Sodium Lactate on Storage Stability and Shelf Life of Red Sour Soup (Hong Suan Tang) Meat Filling

**DOI:** 10.3390/foods15152743

**Published:** 2026-08-04

**Authors:** Jiqing Lei, Menglin Huang, Jianzhi Tian, Jinyong Deng, Xueqin Wang, Rui Wang

**Affiliations:** 1 Department of Food Science and Engineering, Guiyang University, 103 Jianlongdong Road, Guiyang 550005, China; jiqingleigyu@163.com (J.L.); huangmenglin@gyu.edu.cn (M.H.); suiyue@gyu.edu.cn (J.T.); dengjinuong@gyu.edu.cn (J.D.); 2 Guizhou Key Laboratory of New Quality Processing and Storage of Ecological Specialty Food, 103 Jianlongdong Road, Guiyang 550005, China; 3 Guizhou Zhuliang Ecological Technology Food Company Limited, Guiyang Agricultural Products Logistics Park, Guiyang 550200, China; wxueqin2026@163.com

**Keywords:** red sour soup, meat filling, sodium D-isoascorbate, phospholipids, sodium lactate, storage stability, shelf life

## Abstract

Red sour soup dumpling filling, a prepared meat product from Guizhou, China, requires validated shelf-life control strategies for industrial-scale commercialization. This study investigated a composite preservation system of sodium D-isoascorbate, phospholipids, and sodium lactate using a Box–Behnken design, and evaluated its effects on color, lipid oxidation (PV, TBARS), TVB-N, texture, sensory scores, APC, and coliforms during accelerated shelf-life testing. Key indicators were identified by correlation analysis, and shelf life was predicted using log-logistic AFT and temperature-state-dependent kinetic models. The optimized formulation (0.27% sodium D-isoascorbate, 0.11% phospholipids, and 0.05% sodium lactate) significantly inhibited lipid oxidation, retarded color deterioration, maintained texture, and delayed sensory decline. Sensory shelf life (SSL_50_) at 5 °C, −5 °C, and −18 °C extended from 4.44 d to 6.49 d, 16.70 d to 22.90 d, and 108.99 d to 136.76 d, whereas PV-based shelf life extended from 11.0 d to 14.5 d, 15.8 d to 18.8 d, and 124.3 d to 224.3 d. The relative extension of sensory shelf life decreased as storage temperature decreased, whereas PV shelf-life extension surged at low temperatures, indicating a decoupling of lipid oxidation from textural deterioration under frozen conditions. Therefore, shelf-life prediction for dumpling fillings should move away from arbitrary relative thresholds and instead establish sensory-calibrated absolute chemical thresholds, thereby helping align chemical shelf-life indicators with consumer-relevant quality endpoints.

## 1. Introduction

Red sour soup (Hong Suan Tang) is a traditional fermented food of the Miao ethnic group in the southeastern Guizhou region of China. It is typically produced from tomatoes, red chili peppers, and glutinous rice through natural fermentation. This process yields a distinctly acidic flavor system with strong regional characteristics [1,2]. Previous studies have demonstrated that the typical flavor of red sour soup is formed primarily by organic acids such as lactic acid [1,3]. Volatile components including esters, alcohols, and terpenes also contribute to this flavor. In addition, red sour soup contains bioactive components such as lycopene, polyphenols, and carotenoids [1,3,4]. These compounds provide both flavor and nutritional value. At present, red sour soup has been widely applied in food processing, including sour soup fish, sour soup beef, and hot pot condiments. It is progressively developing toward standardization, convenience, and pre-processing.

When combined with meat, red sour soup creates a multidimensional flavor system involving fermented acidity, meat aroma, lipid-derived aroma, and spice notes. The addition of red sour soup during beef cooking significantly reduced pH, increased organic acid content, and increased the relative abundance of esters, acids, and ketones. It also reduced the abundance of off-odor-related aldehydes such as hexanal, thereby improving beef flavor and color [5]. However, such acidic composite systems are generally susceptible to flavor deterioration during storage. In acidified chili products and composite seasoning sauces, prolonged or elevated-temperature storage has been reported to decrease esters and terpenes, and increase acid and peroxide values. Such storage also promotes Strecker degradation, nonenzymatic browning, and oxidation. These reactions ultimately intensify sourness, bitterness, off odors, and volatile sulfur compounds [6,7]. In parallel, prolonged frozen storage is known to accelerate lipid and protein oxidation in meat systems, as evidenced in pork patties and dumpling fillings [8,9,10,11,12]. For instance, in pork dumpling fillings stored at −18 °C, increasing the added fat level from 0 to 30% raised TBARS from 1.60 to 3.29 mg/kg after 180 days [9].

These findings highlight the need for a preservation strategy specifically designed for red-sour-soup-flavored meat filling. In preliminary sensory evaluations conducted by our research group, freshly prepared red-sour-soup-flavored dumpling filling exhibited a pronounced characteristic flavor. However, the intensity of this flavor decreased by more than 70% during 7 days of storage. This result indicates limited flavor retention and a short shelf life. Sodium D-isoascorbate, sodium lactate, and phospholipid-based ingredients have been studied individually or in related combinations. However, no study has evaluated the combined use of sodium D-isoascorbate, sodium lactate, and phospholipids in red sour soup meat filling, nor linked this composite preservation approach to sensory-calibrated shelf-life models.

These three components have potentially complementary functions. Sodium D-isoascorbate has been reported to exhibit reducing activity and to retard lipid oxidation in meat products [13]. Sodium lactate may contribute to microbial inhibition and storage stability through its effects on water availability and metal ion reactions [14]. Phospholipids may regulate lipid dispersion, interfacial stability, and protein–lipid interactions, although they may also serve as oxidation substrates [15]. The combined effects of these components in the acidic red sour soup seasoning matrix therefore require experimental validation.

Accordingly, this study aimed to develop and optimize a three-component preservation system containing sodium D-isoascorbate, sodium lactate, and phospholipids for red-sour-soup-flavored meat filling. Its effects on oxidative stability, microbial stability, texture, color, flavor retention, and sensory quality were evaluated. Single factor experiments and response surface methodology were used to optimize the formulation. Interaction analysis was then performed to determine whether the three components provide complementary or synergistic effects. Finally, shelf life was evaluated by integrating sensory acceptability with physicochemical quality indicators. This study is expected to provide product-specific evidence for flavor retention, quality control, and shelf-life design in red-sour-soup-flavored meat products.

## 2. Materials and Methods

### 2.1. Red Sour Soup Dumpling Filling

The red sour soup dumpling filling was prepared according to the patented formulation provided by Guizhou Zhuliang Ecological Technology Food Co., Ltd., Guiyang, China [1]. The ingredients were combined and homogenized at 20 ± 5 °C for 15 min until a uniform, cohesive filling matrix was obtained.

### 2.2. Response Surface Methodology (RSM) Optimization

**Figure A1:**
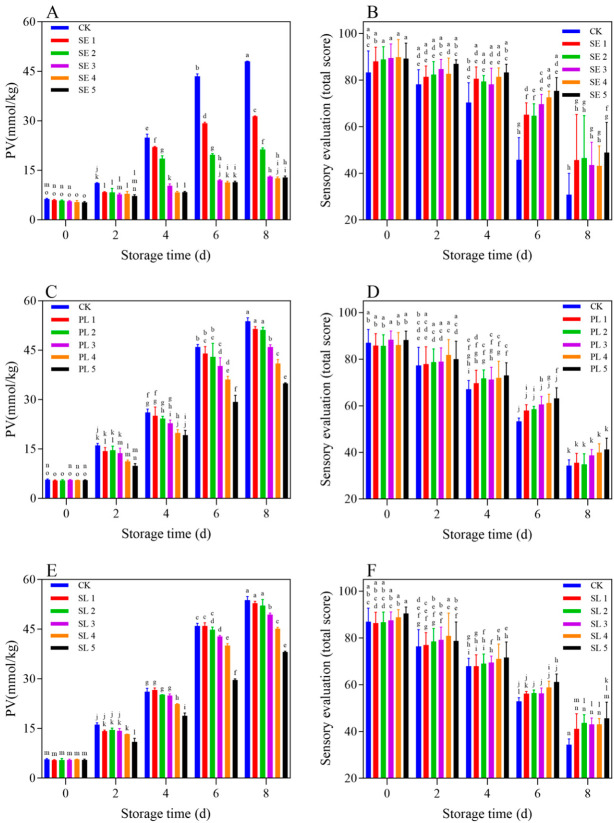
Effects of different concentrations of sodium D-isoascorbate, phospholipids and sodium lactate on peroxide value and sensory scores of meat fillings of red sour soup dumplings under accelerated storage test at 4 °C. (**A**) Peroxide value of meat fillings treated with gradient concentrations of sodium D-isoascorbate; (**B**) sensory evaluation scores of meat fillings treated with gradient concentrations of sodium D-isoascorbate; (**C**) peroxide value of meat fillings treated with gradient concentrations of phospholipids; (**D**) sensory evaluation scores of meat fillings treated with gradient concentrations of phospholipids; (**E**) peroxide value of meat fillings treated with gradient concentrations of sodium lactate; (**F**) sensory evaluation scores of meat fillings treated with gradient concentrations of sodium lactate. CK = untreated control group; SE 1–5 = treatments with different concentrations of sodium D-isoascorbate; PL 1–5 = treatments with different concentrations of phospholipids; SL 1–5 = treatments with different concentrations of sodium lactate. Data are presented as mean ± SD. Different lowercase letters indicate significant differences among different samples. (*p* < 0.05, two-way ANOVA + Tukey’s HSD).

**Figure A2:**
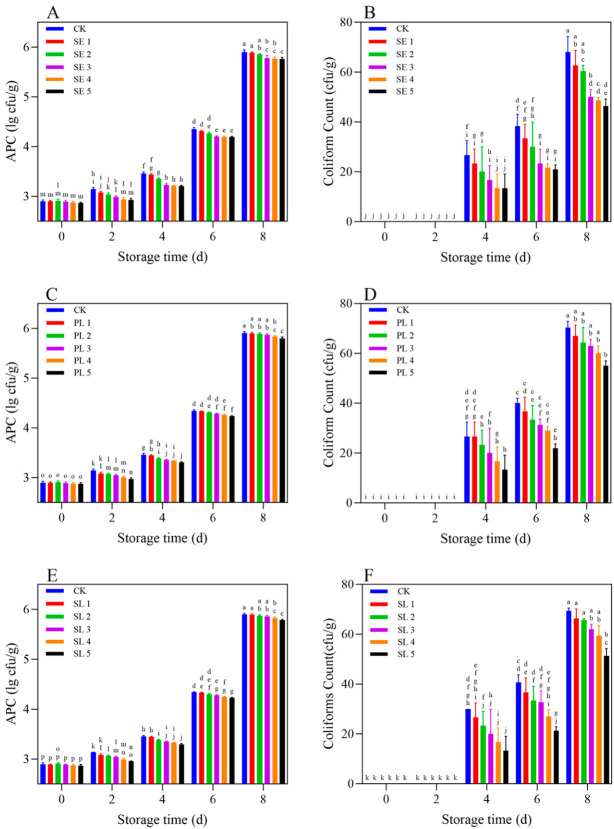
Effects of different concentrations of sodium D-isoascorbate, phospholipids and sodium lactate on total aerobic plate count and coliform counts of meat fillings of red sour soup dumplings under accelerated storage test at 4 °C. (**A**) Total aerobic plate count of meat fillings supplemented with gradient concentrations of sodium D-isoascorbate; (**B**) coliform counts of meat fillings supplemented with gradient concentrations of sodium D-isoascorbate; (**C**) total aerobic plate count of meat fillings supplemented with gradient concentrations of phospholipids; (**D**) coliform counts of meat fillings supplemented with gradient concentrations of phospholipids; (**E**) total aerobic plate count of meat fillings supplemented with gradient concentrations of sodium lactate; (**F**) coliform counts of meat fillings supplemented with gradient concentrations of sodium lactate. No coliforms were detected in samples on day 0 and day 2 of storage. CK = untreated control group; SE 1–5 = treatments with different concentrations of sodium D-isoascorbate; PL 1–5 = treatments with different concentrations of phospholipids; SL 1–5 = treatments with different concentrations of sodium lactate. Data are presented as mean ± SD. Different lowercase letters indicate significant differences among different samples. (*p* < 0.05, two-way ANOVA + Tukey’s HSD).

Based on the single-factor experimental results (Figure A1 and Figure A2), the following variable ranges were selected for the three-factor three-level Box–Behnken design: sodium D-isoascorbate (X_1_) at 0.20–0.40%, phospholipids (X_2_) at 0.08–0.12%, and sodium lactate (X_3_) at 0.04–0.06%. Single-factor experiments showed that coliform counts in all samples remained below the detection limit throughout the observation period, while the other indices began to exhibit significant differences by day 6 of storage at 4 °C. Accordingly, peroxide value (PV), total sensory score, and aerobic plate count (APC) at day 6 of 4 °C storage were selected as the response variables (Y_1_, Y_2_, and Y_3_, respectively). The factor levels and experimental design are presented in Table A1.

Detailed model fitting procedures are described in Appendix C.1.

### 2.3. Accelerated Shelf-Life Testing (ASLT)

The optimized composite preservation system was incorporated into the red sour soup dumpling filling at the levels determined by RSM multi-response optimization to constitute the preservative treatment group (TG). The control group (CK) consisted of the same base filling without the composite preservation system. Both groups were subjected to destructive sampling under two storage regimes: 5 °C (simulating refrigerated distribution) and −5 °C (simulating sub-freezing distribution). At 5 °C, samples were withdrawn at 0, 2, 4, 6, 8, and 10 days; at −5 °C, samples were withdrawn at 0, 4, 8, 12, 16, 20, and 24 days. At each sampling point, the following analyses were performed: color difference (L*, a*, b*), peroxide value (PV), thiobarbituric acid reactive substances (TBARS), total volatile basic nitrogen (TVB-N), aerobic plate count (APC), coliform count, textural profile analysis (TPA), and sensory evaluation. All analyses were conducted immediately after sample withdrawal to minimize post-sampling quality changes.

#### 2.3.1. Color Difference

Color parameters (L*, a*, b*) were measured using a calibrated chroma meter (CR-400, Konica Minolta Co., Ltd., Osaka, Japan) on the sample surface. Measurements were taken at 24 random points per sample. Detailed sample preparation and measurement conditions are described in Appendix C.2.

#### 2.3.2. Sample Preparation for Chemical and Physicochemical Analyses

In total, 30 dumplings were randomly selected, and the meat filling was thoroughly homogenized in a sterile stomacher bag. Aliquots of the homogenized filling were subsequently allocated to each analytical procedure as described below.

#### 2.3.3. Peroxide Value

PV was determined according to the method of do Vale et al. with slight modifications [16]. Lipids were extracted from the meat filling, and the extracted lipid was titrated with sodium thiosulfate standard (Tianjin Kemio Chemical Reagent Co., Ltd., Tianjin, China) solution. The results were expressed as mmol/kg. Full extraction and titration conditions are provided in Appendix C.3.

#### 2.3.4. TBARS

TBARS values were determined according to do Vale et al. with slight modifications [16]. Meat filling was extracted with TCA-EDTA solution, reacted with thiobarbituric acid (TBA; Shanghai Yuanye Bio-Technology Co., Ltd., Shanghai, China), and absorbance was measured at 532 nm. The results were expressed as mg MDA/kg. Detailed reagent concentrations and incubation conditions are described in Appendix C.4.

#### 2.3.5. TVB-N

Determination of total volatile basic nitrogen (TVB-N). TVB-N was determined using an automatic Kjeldahl distillation unit (Model JC-NY20, Qingdao Jingcheng Instrument & Meter Co., Ltd., Qingdao, China) according to Zhang et al. [17]. Meat filling was homogenized with NaCl (Sinopharm Chemical Reagent Co., Ltd., Shanghai, China) solution, distilled with magnesium oxide (Sinopharm Chemical Reagent Co., Ltd., Shanghai, China), and the distillate was titrated with HCl standard solution. The results were expressed as mg N/100 g. Detailed distillation parameters are given in Appendix C.5.

#### 2.3.6. APC and Coliform Counts

APC and coliform counts were determined according to Sajadi et al. with minor modifications [18]. Meat filling was homogenized in sterile saline, serially diluted, and plated on appropriate media. The results were expressed as log CFU/g or log MPN/g. All determinations were performed in triplicate. Incubation conditions and enumeration criteria are detailed in Appendix C.6.

#### 2.3.7. Sensory Evaluation

Sensory evaluation was conducted according to the sensory assessment method for pork dumplings reported by Huang et al. [9], with modifications based on the sensory scoring protocol for sour soup beef described by Liu et al. [19]. A trained panel of 50 assessors (25 female, 25 male, aged 18–25) evaluated all samples throughout the storage period. Each attribute was scored on a 20-point line scale, and overall acceptability was recorded as acceptable/unacceptable for subsequent survival analysis modeling as shown in Table A2. All assessors provided written informed consent prior to participation. Samples were cooked, coded, and presented in randomized order. Panelists provided written informed consent. Detailed panel training, evaluation conditions, and palate cleansing protocols are provided in Appendix C.7.

#### 2.3.8. Textural Properties

Following the method of Pan et al. [20], texture profile analysis (TPA) and shear force determination were performed using a texture analyzer (Stable Micro Systems, Godalming, Co., Ltd., Surrey, UK) equipped with appropriate probes. Instrument settings and probe specifications are detailed in Appendix C.8.

### 2.4. Sensory Shelf Life

Sensory shelf life was modeled using parametric accelerated failure time (AFT) models based on interval-censored rejection data. Model parameterization was performed using a three-point temperature gradient dataset, comprising accelerated shelf-life testing (ASLT) data acquired at 5 °C and −5 °C, supplemented by an additional long-term storage experiment conducted at −18 °C with intensive sampling at 0, 30, 50, 65, 80, 95, 110, and 125 days. Rejection times were treated as interval-censored between the last accepted and first rejected observation. Three candidate distributions (Weibull, lognormal, loglogistic) were evaluated, and the distribution with the lowest AIC value was selected as the final model [21].

The general form of the AFT model was:

(1) $$\log\left(T\right)\;={\;\beta }_{0}+\;\beta_{1}\frac{1}{T_{K}}\;+\;\beta_{2}G\;+\;\beta_{3}\left(\frac{1}{T_{K}}\times G\right)\;+\;\sigma W,$$

where T represents the rejection time (days), $T_{K}$ is the absolute temperature (K), and G is the treatment indicator (0 = CK, 1 = TG); $\beta_{0}$ is the intercept; $\beta_{1}$, $\beta_{2}$ and $\beta_{3}$ are the regression coefficients corresponding to temperature, treatment, and their interaction term, respectively; *σ* is the standard deviation of the error term; and *W* follows a standard normal distribution. Activation energy (*E_a_*) was calculated from the Arrhenius relationship: *E_a_* = *β*_1_ × R, where R = 8.314 J/mol/K. Sensory shelf life was defined as the time corresponding to 50% rejection probability. Detailed model fitting procedures are described in Appendix C.9.

### 2.5. Peroxide Value Shelf Life

The kinetics of peroxide value (PV) during storage were analyzed via temperature-dependent kinetic modeling, with model parameterization performed on a three-point temperature gradient dataset as described in Section 2.4. The evolution of PV as a function of storage time and temperature was described using a Weibull-type plateau model [22]:

(2) $$\mathrm{PV}\left(t,T\right)\;=\;X_{f}\;+\;\left(X_{0}\;-\;X_{f}\right)\exp\left[-k\left(T\right)t^{n}\right],$$

where $\mathrm{PV}\left(t,T\right)\;$ is the peroxide value at time t and temperature T, *t* is the storage time (days), $X_{0}$ and $X_{f}$ represent the initial and asymptotic peroxide values, k(T) is the temperature-dependent rate constant, and n is the kinetic shape parameter. Two temperature dependence functions were evaluated: an exponential temperature model

(3) k(T)=exp(a+βT),

and an Arrhenius model:

(4) $$k(T)\;={\;k}_{\mathrm{ref}}\exp[-E_{a}/R(\frac{1}{T}\;-\;\frac{1}{T_{\mathrm{ref}}}1)],$$

where a and β are empirical parameters, $k_{ref}$ is the rate constant at the reference temperature, $E_{a}$ is the apparent activation energy, and R is the universal gas constant (8.314 J mol^−1^ K^−1^). Two temperature dependence functions (exponential and Arrhenius) were evaluated, with a piecewise formulation for chilling (*T* ≥ −10 °C) and freezing (*T* < −10 °C) regions. Four candidate models were compared using R^2^, RMSE, AIC, AICc, and BIC. Q_10_ was calculated as Q_10_ = k(*T* + 10)/k(*T*). Detailed model fitting procedures are described in Appendix C.10.

### 2.6. Statistical Analysis

All determinations were performed in triplicate, and experimental data are expressed as mean ± standard deviation. Data processing was conducted using Microsoft Excel 2021 (Microsoft Corp., Redmond, WA, USA). Statistical analysis was performed using SPSS Statistics (version 27.0, IBM Corp., Armonk, NY, USA). Response surface methodology (RSM) based on Box–Behnken design (BBD) was employed to optimize the experimental conditions using Design-Expert software (version 13, Stat-Ease Inc., Minneapolis, MN, USA). Differences among group means were evaluated by two-way analysis of variance (ANOVA), followed by Tukey’s multiple range test for post hoc comparison. Statistical significance was set at *p* < 0.05. Spearman rank correlation analysis was conducted using OriginPro 2021 (OriginLab Corp., Northampton, MA, USA). All figures were generated using OriginPro 2021 and GraphPad Prism (version 10, GraphPad Software, San Diego, CA, USA). All measurements were performed in replicates to ensure data reliability: physicochemical properties (*n* = 3), texture profile (*n* = 5), and color difference (*n* = 24).

## 3. Results

### 3.1. Peroxide Value, Sensory Evaluation and Total Viable Count Modeling

**Figure 1:**
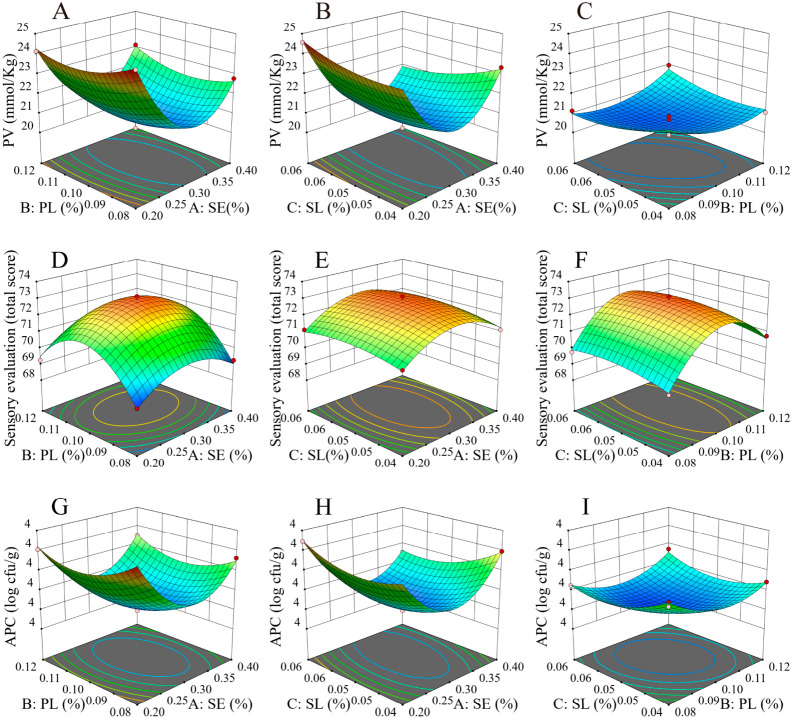
Response surface plots for the interactive effects of Sodium D-isoascorbateand phospholipid, sodium D-isoascorbate and sodium lactate, and phospholipid and sodium lactate on peroxide value, total sensory score, and total viable count. (**A**) The response surface plot of the phospholipid (PL) and sodium D-isoascorbate (SE) interaction for peroxide value; (**B**) the response surface plot of the sodium lactate (SL) and sodium D-isoascorbate (SE) interaction for peroxide value; (**C**) the response surface plot of the sodium lactate (SL) and phospholipid (PL) interaction for peroxide value; (**D**) the response surface plot of the phospholipid (PL) and sodium D-isoascorbate (SE) interaction for total sensory score; (**E**) the response surface plot of the sodium lactate (SL) and sodium D-isoascorbate (SE) interaction for total sensory score; (**F**) the response surface plot of the sodium lactate (SL) and phospholipid (PL) interaction for total sensory score; (**G**) the response surface plot of the phospholipid (PL) and sodium D-isoascorbate (SE) interaction for total viable count; (**H**) the response surface plot of the sodium lactate (SL) and sodium D-isoascorbate (SE) interaction for total viable count; (**I**) the response surface plot of the sodium lactate (SL) and phospholipid (PL) interaction for total viable count. In the 3D surface plots, the color gradient from blue to red indicates an increase in the response value. In the contour plots below, the elliptical lines represent different levels of the response variable.

Under 5 °C storage, second-order polynomial models were fitted for peroxide value (PV) (Equation (5)), sensory evaluation total score (Equation (6)), and total viable count (APC) (Equation (7)), where factors A, B, and C denote the addition levels of sodium D-isoascorbate, phospholipids, and sodium lactate, respectively (Table 1). All three models achieved excellent goodness of fit (R^2^ > 0.980) and were highly significant overall (*p* < 0.001) with non-significant lack of fit (*p* > 0.05), confirming their suitability for prediction and optimization (Table A3). For PV, A (sodium D-isoascorbate), A^2^, and B^2^ were significant (*p* < 0.05), giving the influence order A > C > B (Figure 1A–C). For sensory scores, B, A^2^, and B^2^ were significant (*p* < 0.05), with B^2^ showing the largest sum of squares, giving the influence order B > A > C (Figure 1D–F). For APC, the AC interaction, A, A^2^, B^2^, and C^2^ were significant (*p* < 0.05), giving the order A > B > C (Figure 1G–I).

Multi-response optimization targeting minimum PV, minimum APC, and maximum sensory score yielded an optimal formulation of A = 0.27%, B = 0.11%, C = 0.05%. Under this condition, the predicted values at day 6 were PV = 21.02 mmol/kg, sensory score = 72.4, and APC = 4.22 log CFU/g, achieving the overall optimum. To validate the model, three independent verification experiments were conducted at the optimal formulation. The experimental values were compared with model predictions using Student’s *t*-test. No significant difference was observed between the predicted and actual values (*p* > 0.05), confirming model reliability.

### 3.2. Color Change During Accelerated Shelf-Life Testing

**Figure 2:**
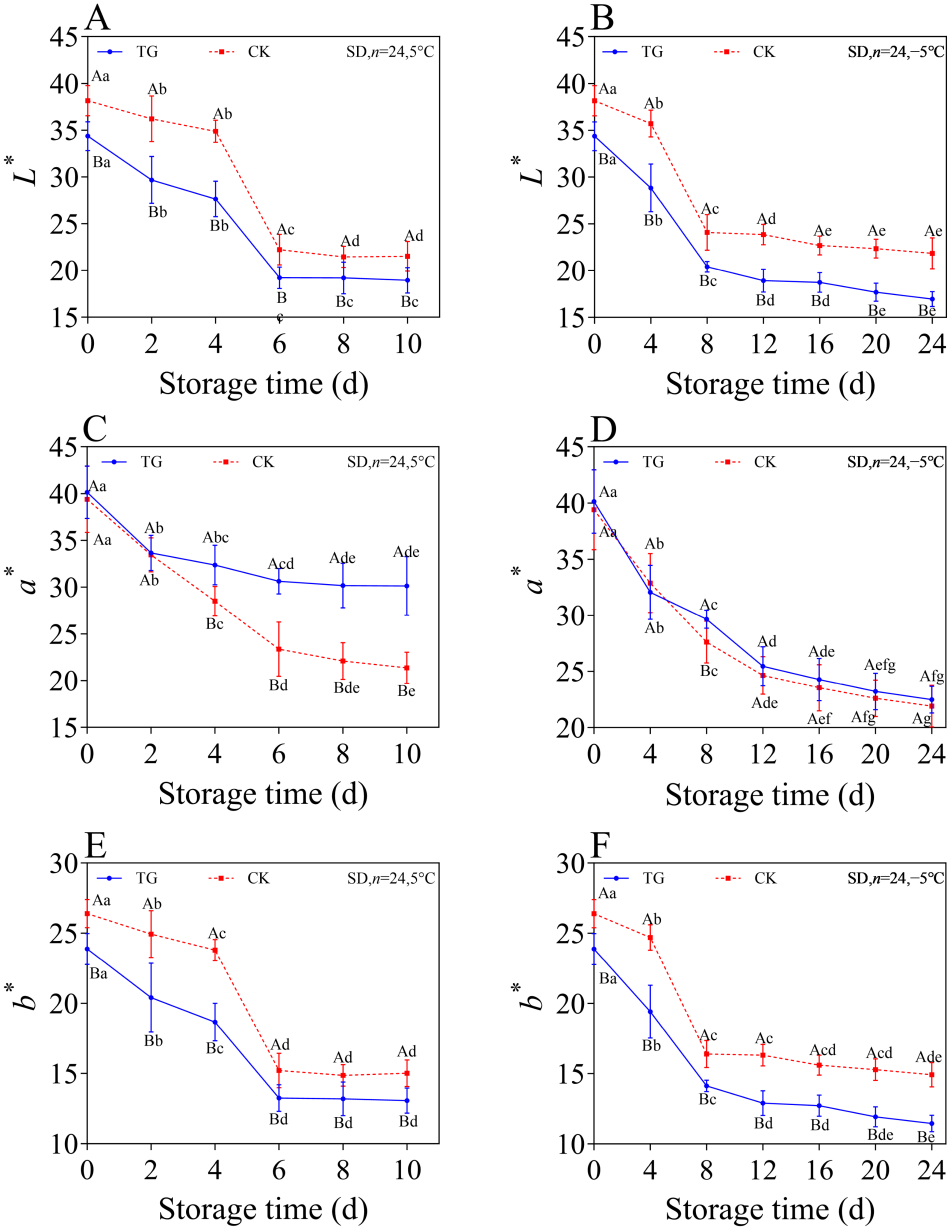
The effects of compound preservatives on the color of sour soup dumpling meat filling under different storage temperatures. (**A**) The L* values of the CK and TG of sour soup dumpling meat filling in the destructive test at 5 °C; (**B**) the L* values of the CK and TG of sour soup dumpling meat filling in the destructive test at −5 °C; (**C**) the a* values of the CK and TG of sour soup dumpling meat filling in the destructive test at 5 °C; (**D**) the a* values of the CK and TG of sour soup dumpling meat filling in the destructive test at −5 °C; (**E**) the b* values of the CK and TG of sour soup dumpling meat filling in the destructive test at 5 °C; (**F**) the b* values of the CK and TG of sour soup dumpling meat filling in the destructive test at −5 °C. The data are presented as mean ± SD (*n* = 24). The uppercase letters indicate inter-group differences at identical storage days, and the lowercase letters indicate intra-group differences over storage time (*p* < 0.05, two-way ANOVA + Tukey’s HSD).

During accelerated destructive storage at 5 °C and −5 °C, the L*, a*, and b* values of the red sour soup dumpling filling all decreased progressively over time. For lightness (L*), the TG showed lower initial values than the CK at both temperatures, and the gap between the two groups widened with prolonged storage (Figure 2A,B). Redness (a*) exhibited the most informative changes regarding the color-stabilizing efficacy of the composite preservation system. Under 5 °C storage, the TG maintained higher a* values than the CK overall. At −5 °C, the a* values of the CK and TG were comparable in the early stage, but from day 4 onward CK became significantly lower than TG and continued to decline (Figure 2C,D). For yellowness (b*), the CK was higher than the TG at both temperatures, with both groups showing synchronous b* decline over storage (Figure 2E,F).

### 3.3. Lipid Oxidation and TVB-N During Accelerated Shelf-Life Testing

**Figure 3:**
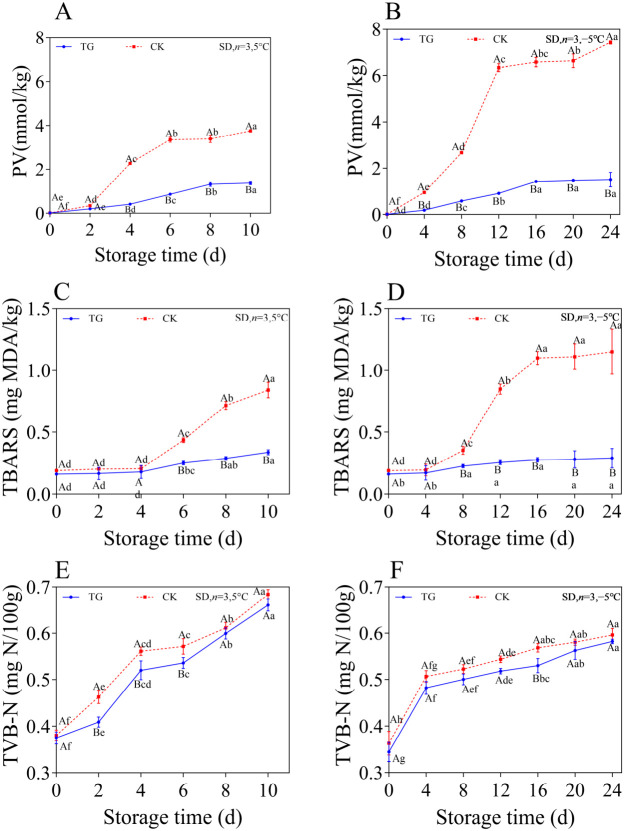
The effects of compound preservatives on the peroxide value, malondialdehyde content and total volatile basic nitrogen of sour soup dumpling meat filling at different temperatures. (**A**) The peroxide value of the samples in the destructive test at 5 °C; (**B**) the peroxide value of the samples in the destructive test at −5 °C; (**C**) the malondialdehyde content of the samples in the destructive test at 5 °C; (**D**) the malondialdehyde content of the samples in the destructive test at −5 °C; (**E**) the total volatile basic nitrogen content of the samples in the destructive test at 5 °C; (**F**) the total volatile basic nitrogen content of the samples in the destructive test at −5 °C. The data are presented as mean ± SD (*n* = 3). The uppercase letters indicate inter-group differences at identical storage days, and the lowercase letters indicate intra-group differences over storage time (*p* < 0.05, two-way ANOVA + Tukey’s HSD).

During accelerated destructive storage, the peroxide value (PV) and thiobarbituric acid reactive substances (TBARS) of the red sour soup dumpling filling increased significantly over storage time, exhibiting a typical lipid oxidation deterioration trend. Compared with the CK, the TG significantly suppressed the elevation of PV and TBARS at both storage temperatures. At 5 °C, it reduced PV by approximately 70 to 80% (Figure 3A) and TBARS by approximately 44 to 57% (Figure 3C) compared with the CK during days 6 to 10. At −5 °C, it reduced PV by approximately 75 to 85% (Figure 3B) and TBARS by approximately 48 to 73% (Figure 3D) compared with the CK during days 8 to 24.

In contrast to the lipid oxidation indices, total volatile basic nitrogen (TVB-N) showed a gradual upward trend at both storage temperatures. At 5 °C, the TG significantly inhibited TVB-N accumulation during days 0 to 6. This difference disappeared during days 8 to 10 (Figure 3E). At −5 °C, no significant difference in TVB-N was observed between the TG and CK throughout the storage period (Figure 3F).

### 3.4. Textural Properties During Accelerated Shelf-Life Testing

**Figure 4:**
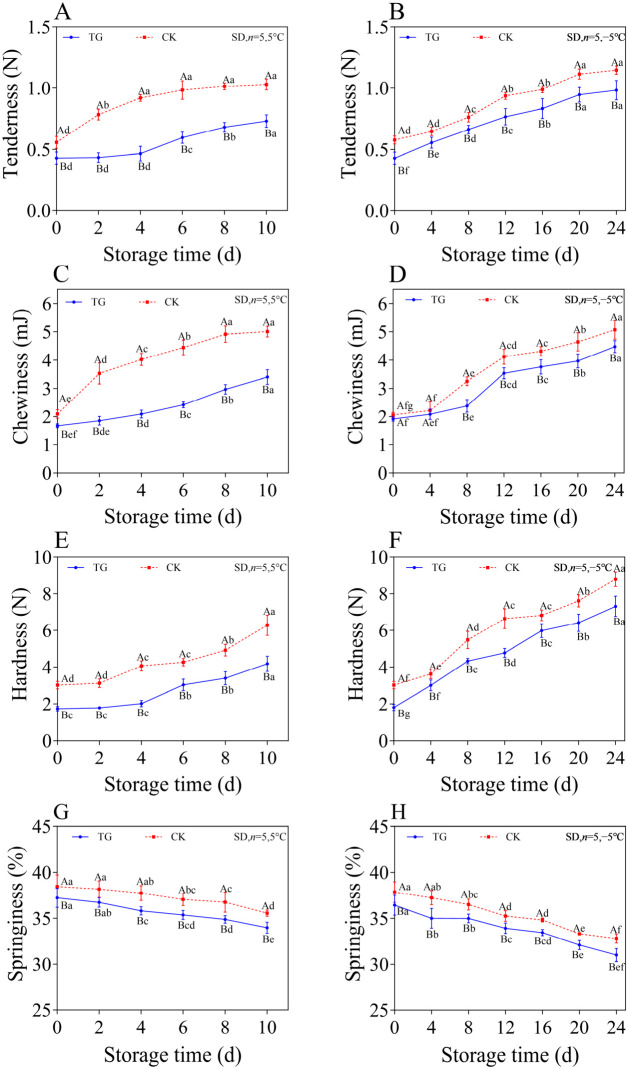
The effects of compound preservatives on the tenderness, chewiness, hardness and springiness of sour soup dumpling meat filling at different temperatures. (**A**) The tenderness of the samples in the destructive test at 5 °C; (**B**) the tenderness of the samples in the destructive test at −5 °C; (**C**) the chewiness of the samples in the destructive test at 5 °C; (**D**) the chewiness of the samples in the destructive test at −5 °C; (**E**) the hardness of the samples in the destructive test at 5 °C; (**F**) the hardness of the samples in the destructive test at −5 °C; (**G**) the springiness of the samples in the destructive test at 5 °C; (**H**) the springiness of the samples in the destructive test at −5 °C. The data are presented as mean ± SD (*n* = 5). The uppercase letters indicate inter-group differences at identical storage days, and the lowercase letters indicate intra-group differences over storage time (*p* < 0.05, two-way ANOVA + Tukey’s HSD).

During accelerated destructive storage at 5 °C and −5 °C, the shear force, chewiness, and hardness of both groups increased significantly over time (*p* < 0.05), while springiness decreased significantly (*p* < 0.05) (Figure 4A–F). The TG significantly retarded the increase in shear force, chewiness, and hardness at both storage temperatures (*p* < 0.05).

Notably, the protective effect of the TG on springiness exhibited a differential response (Figure 4G,H). At 5 °C, no significant difference in springiness decline was observed between the TG and CK (*p* > 0.05). At −5 °C, the TG showed significantly lower springiness than the CK at day 4 (*p* < 0.05), but the between-group difference became non-significant from day 8 through day 20, and by the end of storage (day 24) the TG exhibited slightly lower springiness than the CK.

### 3.5. Sensor Evaluation During Accelerated Shelf-Life Testing

**Figure 5:**
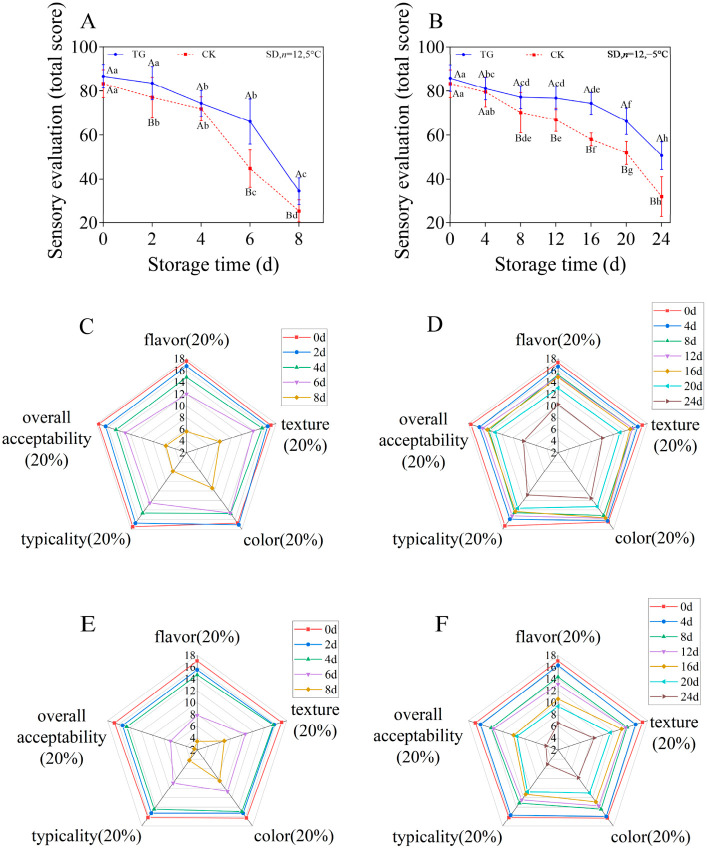
The effects of compound preservatives on the sensory evaluation properties of red sour soup dumpling meat filling at different temperatures. (**A**) The total sensory score of the samples in the destructive test at 5 °C; (**B**) the total sensory score of the samples in the destructive test at −5 °C; (**C**) a sensory radar chart of the TG in the destructive test at 5 °C; (**D**) a sensory radar chart of the TG in the destructive test at −5 °C; (**E**) a sensory radar chart of the CK in the destructive test at 5 °C; (**F**) a sensory radar chart of the CK in the destructive test at −5 °C. The data are presented as mean ± SD (*n* = 12). The uppercase letters indicate inter-group differences at identical storage days, and the lowercase letters indicate intra-group differences over storage time (*p* < 0.05, two-way ANOVA + Tukey’s HSD).

During accelerated destructive storage, the sensory evaluation total score of the red sour soup dumpling filling declined significantly with prolonged storage time, and the rate of deterioration was closely related to storage temperature. At 5 °C, the CK showed a rapid decrease in sensory total score. The TG maintained significantly higher scores than the CK on days 2, 6, and 8. At the end of storage, the score of the TG was approximately 30% higher than that of the CK (Figure 5A). At −5 °C, both groups showed a gradual decline in total score. However, the TG remained relatively stable. Its scores were significantly higher than those of the CK from day 8 onward. At day 24, the score of the TG was approximately 70% higher than that of the CK (Figure 5B).

Radar chart analysis further revealed that all five sub-indices—flavor, texture, color, typicality, and overall acceptability—contracted collectively over storage time, indicating that the various sensory dimensions did not deteriorate independently but were driven by a common quality degradation mechanism. The contraction of the radar area was much slower at −5 °C than at 5 °C. The TG maintained a larger radar area than the CK at the late storage stage under both temperatures. (Figure 5C–F).

### 3.6. Microbial Growth During Accelerated Shelf-Life Testing

During accelerated destructive storage, the microbial indicators of the red sour soup dumpling filling exhibited significant temperature dependence (Table 2). At 5 °C, the APC of both the CK and TG increased continuously over storage time and reached unacceptable levels by day 8. The APC of the TG remained lower than that of the CK throughout storage. At −5 °C, the APC of both groups showed an overall downward trend and stabilized.

At 5 °C, the coliform counts of both groups increased gradually over time. The composite preservative reduced coliform counts by approximately 51 to 73% compared with the CK during days 4 to 8. At −5 °C, low temperature markedly suppressed microbial activity. Coliforms were only detected in the CK on day 24 of storage, while no coliforms were detected in the TG.

### 3.7. Correlation Analysis

**Figure 6:**
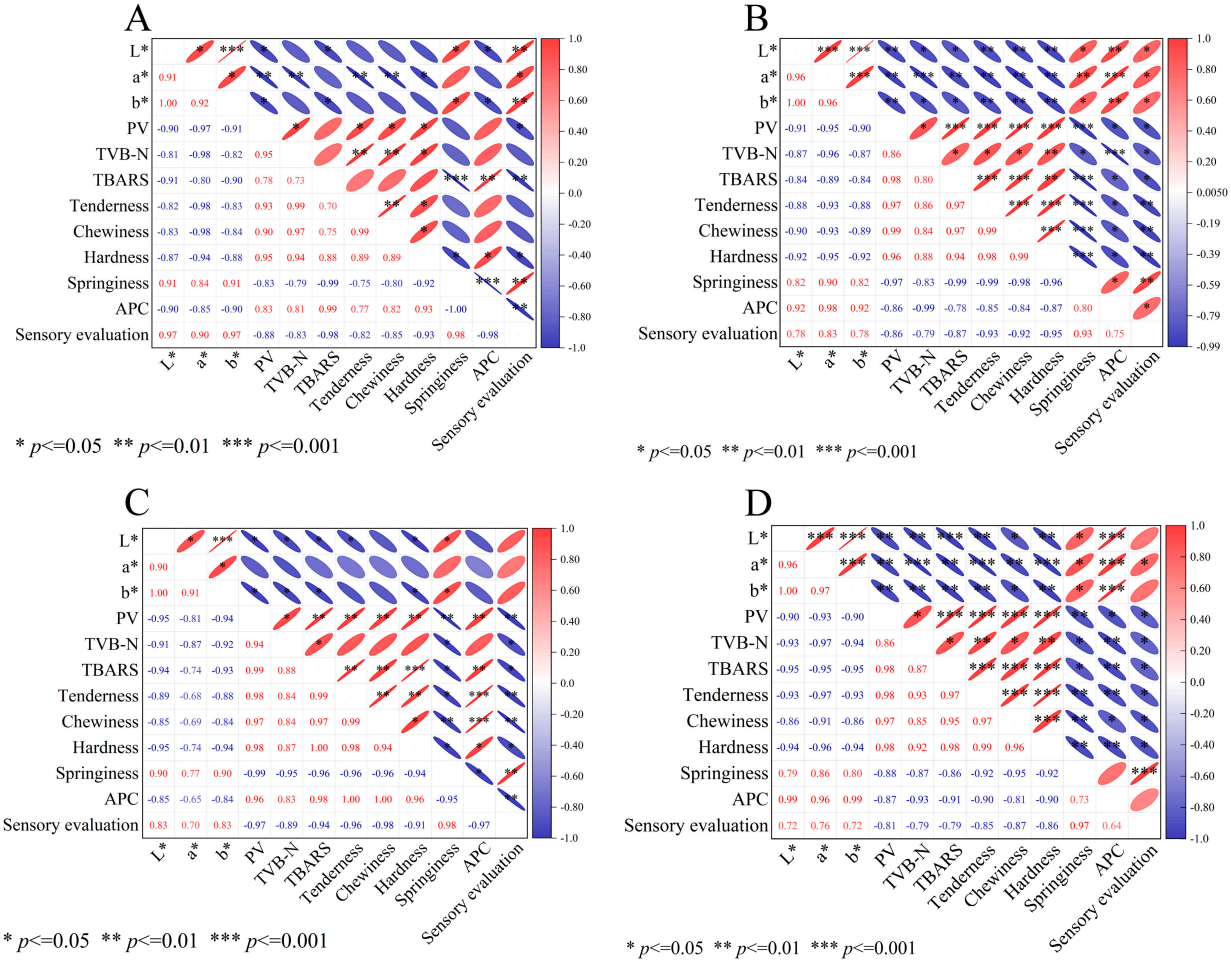
Correlation heatmaps of multiple quality indicators of the sour soup dumpling meat filling at different temperatures. (**A**) A correlation heatmap of the physicochemical, color, texture, total sensory and microbial indicators of meat filling in the CK under destructive storage at 5 °C; (**B**) a correlation heatmap of the physicochemical, color, texture, total sensory and microbial indicators of meat filling in the CK under destructive storage at −5 °C; (**C**) a correlation heatmap of the physicochemical, color, texture, total sensory and microbial indicators of meat filling in the TG under destructive storage at 5 °C; (**D**) a correlation heatmap of the physicochemical, color, texture, total sensory and microbial indicators of meat filling in the TG under destructive storage at −5 °C. The heatmaps reflect the correlations among the physicochemical indicators, color parameters, texture properties, total sensory score and microbial indicators of the meat filling. Pearson correlation analysis was performed to evaluate the correlation between each pair of indicators, with the correlation coefficient denoted as * *p* < 0.05, ** *p* < 0.01, *** *p* < 0.001.

Pearson correlation analysis was performed on the color indices (L*, a*, b*), oxidative markers (PV, TVB-N, TBARS), textural attributes (tenderness, chewiness, hardness, springiness), and microbiological indicators (APC) to elucidate the intrinsic quality deterioration networks under different storage temperatures (Figure 6). The correlation architectures revealed fundamental differences between the CK and TG.

Under storage at 5 °C, the sensory scores and springiness in the CK group were significantly negatively correlated with PV, TVB-N, TBARS, and APC (R > 0.9, *p* < 0.05). In contrast, APC, PV, and TVB-N showed significant positive correlations with hardness, chewiness, and tenderness (R > 0.9, *p* < 0.05). After the TG combined treatment, the correlation network among quality attributes became more compact and internally consistent. However, the correlations between TVB-N and hardness, chewiness, and tenderness were weakened (R < 0.9, *p* < 0.05), whereas the correlations between TBARS and these textural parameters increased markedly and approached 1.0.

Under superchilling storage at −5 °C, sensory scores and springiness remained significantly negatively correlated with PV, TVB-N, TBARS, and APC. In contrast to the pattern observed at 5 °C, APC became significantly negatively correlated with PV, TVB-N, TBARS, hardness, chewiness, and tenderness (R < −0.79, *p* < 0.05). No obvious difference was observed between the CK and TG groups.

### 3.8. Shelf-Life Estimation and Validation

As the correlation structures differed across storage conditions, shelf life was estimated using two complementary models. A log-logistic accelerated failure time model, referred to as the AFT model, was fitted to sensory accept or reject records containing interval-censored and right-censored observations. A temperature-state-dependent kinetic model was used to describe peroxide value accumulation at 5 °C, −5 °C, and −18 °C. The estimated parameters for the AFT model were detailed in Table A4.

Among the candidate distributions evaluated for the sensory AFT model, the log-logistic distribution provided the best fit, with an Akaike information criterion value of 479.03. This value was lower than those of the lognormal distribution and Weibull distribution, which had Akaike information criterion values of 483.51 and 503.40, respectively (Table A5).

**Figure 7:**
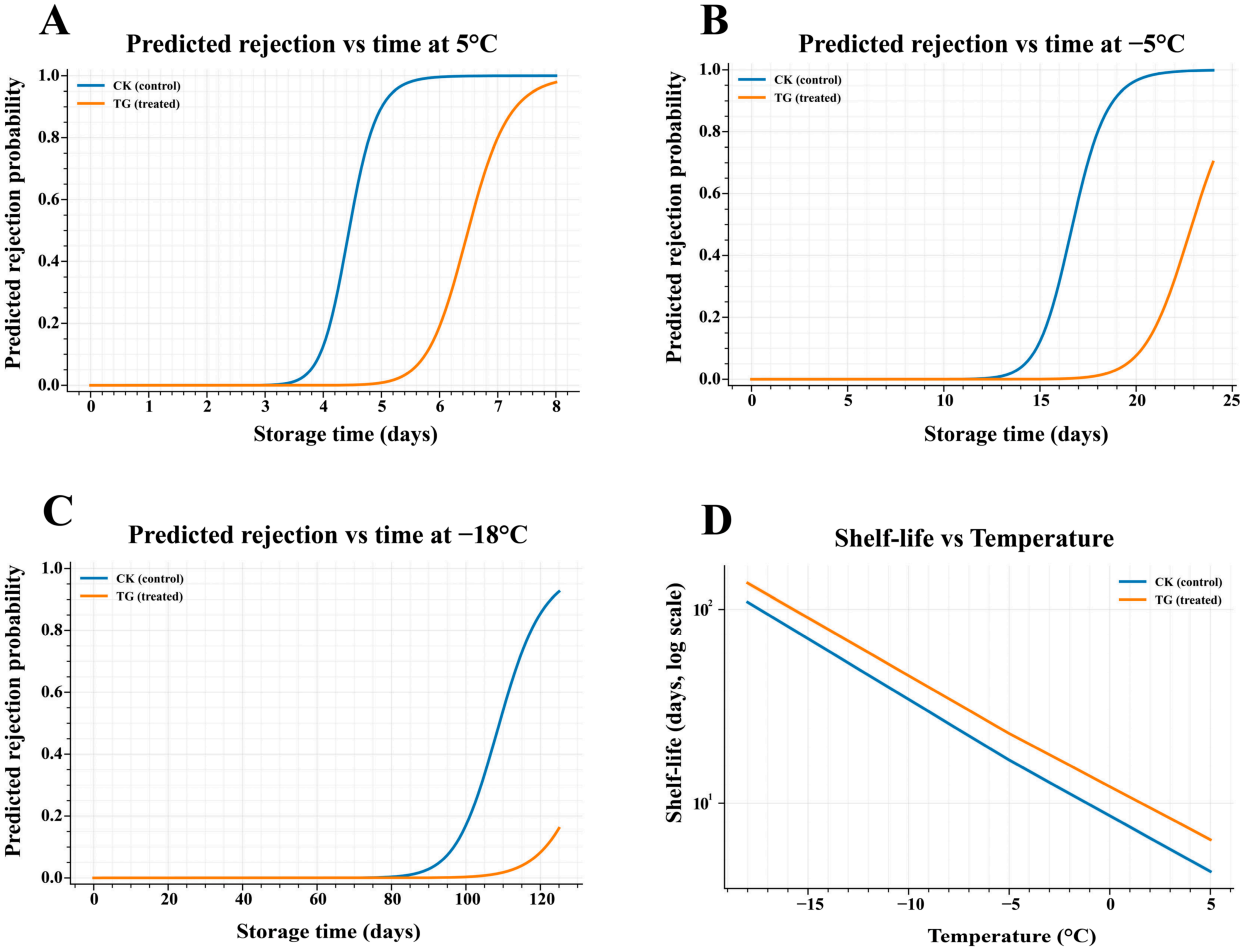
An evaluation of the effects of sodium D-isoascorbate, phospholipids and sodium lactate on the sensory shelf life of sour soup dumplings based on survival analysis. (**A**) The predicted sensory rejection probability against storage time at 5 °C; (**B**) the predicted sensory rejection probability against storage time at −5 °C; (**C**) the predicted sensory rejection probability against storage time at −18 °C; (**D**) the log-scale predicted sensory shelf life (SSL_50_) as a function of storage temperature. The blue lines represent the CK control group, and the orange lines represent the TG composite anti-oxidant treatment group.

The predicted sensory shelf lives at 50% rejection probability level (SSL_50_) were 4.44 ± 0.06 days for the CK and 6.49 ± 0.07 days for the TG at 5 °C. At −5 °C, the corresponding SSL_50_ was 16.70 ± 0.22 days and 22.90 ± 0.15 days. At −18 °C, they were 108.99 ± 1.38 days and 136.76 ± 2.23 days, respectively (Table 3). Thus, the TG treatment extended sensory shelf life by 46%, 37%, and 25% at 5 °C, −5 °C, and −18 °C, respectively (Table 3). The sensory rejection probability curves shifted toward longer shelf life for the TG at all temperatures, but the curves gradually converged as the storage temperature decreased; the relative benefit of the preservation treatment was reduced under deep frozen conditions (Figure 7).

**Figure 8:**
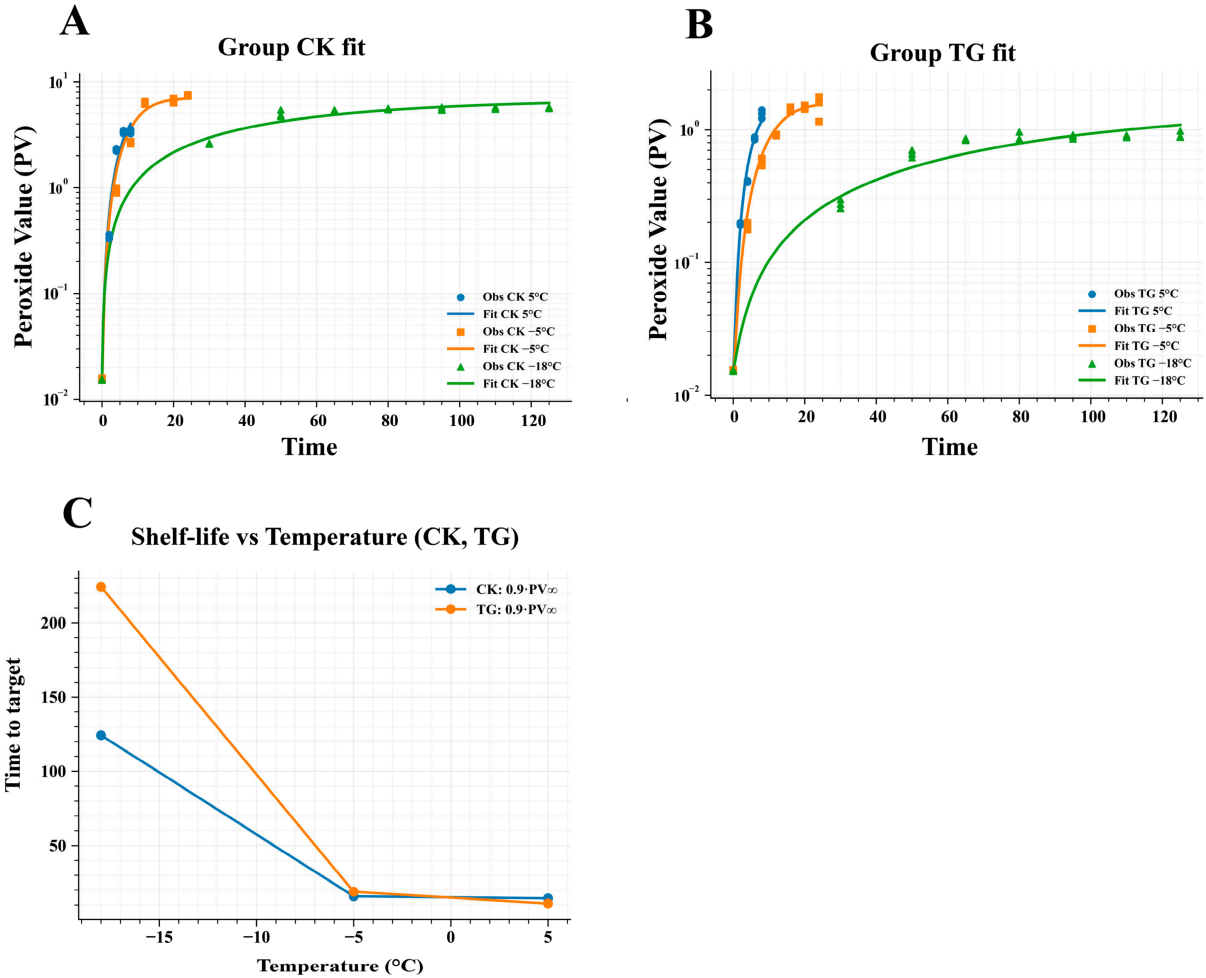
PV kinetic fitting and oxidation-limited shelf life of CK (control) and TG (preservative treatment). (**A**): Observed experimental PV points (Obs) and corresponding model fitted curves (Fit) of control group (CK) at 5 °C, −5 °C and −18 °C; (**B**): observed experimental PV points (Obs) and corresponding model fitted curves (Fit) of preservative treatment group (TG) at 5 °C, −5 °C and −18 °C; (**C**): predicted shelf life reaching relative threshold of 0.9 PV∞ plotted against storage temperature for CK and TG.

Among the four candidate models evaluated by AIC, R^2^, and RMSE, the expT_piecewise_n model provided the best fit for both the CK (AIC = −62.20, R^2^ = 0.952, RMSE = 0.53) and TG (AIC = −250.80, R^2^ = 0.945, RMSE = 0.11) (Table A6). Arrhenius-type models were retained for kinetic interpretation and shelf-life prediction owing to their marginally superior statistical performance (Table A7). The initial PVs were nearly identical between groups (0.0154 mmol/kg for both CK and TG); the asymptotic peroxide value, denoted as PV∞, was substantially lower in the TG than in CK, at 1.589 and 7.014 mmol kg^−1^, respectively (Figure 8A and Figure 8B).

Applying a relative threshold of 0.9·PV∞ to the kinetic model predicted PV-based shelf lives of 11.0 days for the CK and 14.5 days for the TG at 5 °C. At −5 °C, 1 the corresponding values were 5.8 days and 18.8 days. At −18 °C, they were 124.3 days and 224.3 days, respectively (Figure 8C and Table 3).

## 4. Discussion

Changes in color, lipid oxidation, and myoglobin oxidation during meat storage and processing form a tightly coupled deterioration cascade. Meat color is primarily governed by interconversion among myoglobin redox forms: the conversion of oxymyoglobin to metmyoglobin decreases a* values and promotes browning, whereas maintaining a high proportion of oxymyoglobin improves color stability [23,24]. In turn, peroxyl radicals and reactive aldehydes formed during lipid oxidation further promote myoglobin oxidation. This creates a positive feedback loop of quality deterioration [25,26].

The components of the composite preservation system interfere with this coupled cascade at different points and therefore act synergistically. Sodium lactate can chelate pro-oxidative Fe^2+^ and reduce its catalytic role in both lipid and myoglobin oxidation [27]. It may also sustain metmyoglobin-reducing activity, lactate dehydrogenase activity, and NADH levels through the lactate–lactate dehydrogenase–NADH system. This promotes the reduction of metmyoglobin to nitrosomyoglobin and delays browning, although it may slightly darken the apparent color [28,29]. Ascorbic acid can reduce ferrylmyoglobin [MbFe(IV) = O] to ferric metmyoglobin, interrupting the oxidative chain reaction [30,31]. It also quenches radicals by electron donation, suppresses lipid autoxidation propagation, and promotes nitrosomyoglobin formation [26,32,33]. Consistently, Shang reported that sodium erythorbate reduced metmyoglobin and radical accumulation in Cantonese sausage. The composite preservation system therefore retarded both lipid and myoglobin oxidation and preserved color and flavor [34].

The present results are highly consistent with these mechanisms. Under refrigerated storage at 5 °C, the composite preservation system decreased L* and b* values and significantly delayed the decline in a* during the middle and late storage stages. At the same time, PV and TBARS were significantly reduced, and the differences between the treatments became more evident as storage progressed. This indicates that the system restrained both the initiation and the propagation of the oxidative chain reaction. Overall, the composite preservation system of sodium lactate and D-ascorbic acid effectively weakened the coupled deterioration among color change, lipid oxidation, and myoglobin oxidation by maintaining a reducing environment and interrupting radical chain reactions [34].

However, under superchilling at −5 °C, the composite preservation system still lowered PV and TBARS significantly but failed to improve a* values. Previous studies have shown that under freezing conditions, metmyoglobin accumulation, TBARS formation, protein carbonylation, and sulfhydryl loss are generally slowed, but their correlations remain [35]. In this temperature range, ice-crystal-induced cell rupture, freeze concentration in the unfrozen phase, and local enrichment of pro-oxidants and oxidative enzymes may all accelerate protein oxidation [8,36,37]. Although protein oxidation is not the direct determinant of color, it can amplify overall color differences by altering myofibrillar spacing, water-holding status, and light-scattering behavior [38]. Thus, under superchilling conditions, the composite preservation system may have failed to preserve a* because it could not effectively suppress ice-crystal damage, drip loss, and the associated optical changes. In addition, sodium lactate and ascorbic acid did not significantly reduce TVB-N. This suggests limited interference with the pathways of volatile basic nitrogen formation [36,39].

With respect to texture regulation, the phospholipid component of the composite preservation system likely played a major role. Phospholipids have been reported to interact with myosin through hydrophobic interactions and hydrogen bonding, promoting the transition of α-helices to β-sheets or random coils and exposing hydrophobic sites, thereby improving emulsifying properties, rheology, and gel strength [40,41,42]. Xia et al. reported that repeated freeze–thaw cycles induced denaturation and aggregation of myofibrillar proteins, leading to significantly increased hardness and chewiness and decreased springiness [43]. In meatball and surimi systems, phospholipids or lecithin have been shown to enhance water retention, promote a more homogeneous gel network, and mitigate ice-crystal-induced structural damage [44,45,46]. The present results follow this general pattern. The shear force, hardness, and chewiness of the red sour soup dumpling meat filling generally increased with storage time. The composite preservation system significantly delayed the increases in shear force and hardness at both 5 and −5 °C. This effect is likely attributable mainly to the ability of soybean phospholipids to unfold protein networks, regulate interfacial properties, and improve emulsification. D-ascorbic acid and sodium lactate may have indirectly protected myofibrillar protein conformation and water-holding capacity by suppressing lipid oxidation and carbonylation [34]. Nevertheless, under −5 °C, the composite preservation system reduced hardness but did not alter its overall increasing trend. This suggests that ice-crystal-induced protein structural disruption remained the dominant driver of texture deterioration at this temperature [47,48].

The sensory results further indicate that the dominant failure mechanism varies across temperature ranges. Limbo et al. reported that total sensory score and overall acceptability are the most reliable indicators of shelf-life termination and are significantly associated with APC and lipid oxidation [49]. This is consistent with the findings at 5 °C in the present study. At 5 °C, the TG samples remained sensorially acceptable up to day 24, whereas the CK samples approached the rejection threshold; sensory scores and springiness in the CK group were significantly negatively correlated with PV, TVB-N, TBARS, and APC. However, the relative benefit of the TG at −5 °C was smaller than that at 5 °C. Moreover, APC decreased continuously at subzero storage temperatures, and correlation analysis revealed that APC became significantly negatively correlated with PV, TVB-N, TBARS, hardness, chewiness, and tenderness. These observations suggest that at temperatures below freezing, low temperature itself provides an effective barrier against microbial proliferation, thereby attenuating the antimicrobial contribution of the composite preservation system relative to refrigeration. These findings align with Yi et al. [48], who reported that microbial proliferation, protein oxidation, water migration, and texture deterioration are inter-related in chicken breast stored at low temperatures [48].

Given this temperature dependence, the present study combined survival analysis with a temperature- and state-dependent kinetic model to characterize shelf life. In this framework, sensory analysis describes the time distribution of consumer rejection, whereas the PV kinetic model describes the evolution of a specific oxidation-related physicochemical index at a given temperature [21,50,51].

At 5 °C, the accelerated failure time model predicted a median SSL50 of only 4.4–6.5 days, whereas the PV model predicted 11.0–14.5 days, a difference of about 2.5-fold. This indicates that under refrigeration, consumer rejection is jointly driven by microbial proliferation, protein degradation, accumulation of volatile metabolites, color deterioration, and early oxidation, whereas the PV endpoint mainly reflects oxidation itself [51,52]. Therefore, in this temperature range, a single oxidation endpoint is better interpreted as a process-characterization index than as a direct substitute for consumer-relevant shelf life.

This mismatch between sensory and oxidation endpoints became more pronounced at −5 and −18 °C. At −5 °C, the TG samples were sensorially rejected when they had reached only 24% of their asymptotic PV. This indicates that products may fail prematurely because of moisture migration, localized freezing, and textural deterioration even when lipid oxidation remains low [48]. At −18 °C, the failure mechanism can no longer be explained by the simple logic that slower oxidation necessarily means longer shelf life. Similar mismatches have been reported. Beef stored at −18 °C showed the best chemical and microbiological stability yet exceeded the consumer acceptability threshold for shear force [53]. Wang et al. likewise emphasized that kinetic models based on TBARS and TVB-N are better suited to predicting quality evolution than to defining a universal consumer rejection shelf life [54].

Comparable composite preservation systems have been reported in other meat products. An ε-polylysine, sodium lactate, and D-sodium ascorbate system inhibited myoglobin oxidation and maintained color in refrigerated tuna [55]. A sodium erythorbate and lactate system showed similar anti-oxidant and antimicrobial activity [56]. A Lactiplantibacillus plantarum hydrogel combined with gallic acid and ice-temperature storage extended the shelf life of yak meat to 16 to 20 days [57]. An ε-polylysine, tea polyphenols, and nisin solution combined with a chitosan film extended the shelf life of chilled venison to 25 days [58]. These studies confirm the value of multitarget preservation. However, most of them were evaluated at a single storage temperature.

In contrast, the present system was evaluated at 5, −5, and −18 °C, and its benefit was shown to be temperature-dependent. Under subzero storage, texture rather than microbial or oxidative deterioration became the key factor limiting sensory acceptability. A major limitation of the present system is therefore the lack of cryoprotective or structural-protection components. Future studies should optimize the ratios of the three components and further screen polysaccharides, proteins, or hydrogel-based ingredients with ice-crystal-inhibiting and water-holding properties.

## 5. Conclusions

In this study, a composite preservation system containing sodium D-isoascorbate (0.27%), phospholipids (0.11%), and sodium lactate (0.05%) was developed for red sour soup dumpling meat filling. The treatment effectively preserved color, inhibited lipid oxidation and microbial proliferation, and delayed textural hardening and sensory deterioration during storage. The pattern of quality deterioration was strongly temperature-dependent. Microbial growth and lipid oxidation jointly dominated quality loss at 5 °C. Although the composite preservation system improved product quality under both refrigerated and superchilled conditions, it did not alter the fundamental temperature-associated deterioration pattern. Shelf-life predictions based on sensory evaluation and PV kinetics indicated that the composite preservation system significantly delayed overall quality deterioration and extended shelf life at 5, −5, and −18 °C, with a greater relative benefit under refrigerated storage than under superchilled storage. Moreover, the PV-based shelf life was consistently longer than SSL_50_, and PVs at sensory rejection remained far below the oxidation limit specified by the Chinese national standard. This finding suggests that sensory failure was primarily triggered by nonoxidative factors rather than by the attainment of an oxidative endpoint. These results provide a practical, product-specific strategy for shelf-life management of red-sour-soup-flavored meat fillings. However, further validation under real-world commercial storage and distribution conditions is needed to confirm the practical applicability of the developed preservation system.

## Figures and Tables

**Table 1:** Response surface models for different indicators.

No.	Equation	
1	$\mathrm{PV}\left(\mathrm{mmol}/\mathrm{kg}\right)\;=\;75.39\;-\;144.84A\;-\;433.93B\;-\;390.06C\;+\;116.75\mathrm{AB}\;-\;528.35\mathrm{AC}$ $\;+\;2058.50\mathrm{BC}\;+\;252.03A^{2}\;+\;1474.73B^{2}\;+\;3296.92C^{2}$,	(5)
2	Sensory Scores=−4.29+70.3A+1103.83B+377.67C+47.5AB+30.00AC − 150BC − 123.42A^2^ − 5435.42B^2^ − 3591.67C^2^,	(6)
3	$\mathrm{APC}\left(\log\frac{\mathrm{cfu}}{g}\right)=4.21\;-\;0.0047A\;-\;0.0012B\;-\;0.0018C+0.0014\mathrm{AB}\;-\;0.0046\mathrm{AC}$ + 0.0024BC + 0.0201A^2^ + 0.0084B^2^ + 0.0061C^2^,	(7)

**Table 2:** Aerobic Plate Count (APC) and Coliform Counts in Samples During Accelerated Shelf-Life Storage.

Storage Temperature/°C	Storage Time/d	APC (log cfu/g)		Coliform Count (cfu/g)	
		CK	TG	CK	TG
5	0	2.88 ± 0.04 ^Ae^	2.75 ± 0.02 ^Bd^	-	-
	2	3.11 ± 0.00 ^Ad^	2.78 ± 0.02 ^Bd^	-	-
	4	3.44 ± 0.01 ^Ac^	2.85 ± 0.02 ^Bc^	23.33 ± 4.71 ^Ac^	6.67 ± 4.71 ^Bc^
	6	4.31 ± 0.01 ^Ab^	4.15 ± 0.01 ^Bb^	37.00 ± 3.74 ^Ab^	10.00 ± 0.00 ^Bb^
	8	5.88 ± 0.013 ^Aa^	5.72 ± 0.03 ^Ba^	68.67 ± 4.19 ^Aa^	33.67 ± 4.64 ^Ba^
−5	0	2.88 ± 0.04 ^Aa^	2.75 ± 0.01 ^Ba^	-	-
	4	2.52 ± 0.03 ^Aab^	2.46 ± 0.02 ^Bb^	-	-
	8	2.38 ± 0.06 ^Ab^	2.21 ± 0.05 ^Bc^	-	-
	12	2.34 ± 0.05 ^Ab^	2.13 ± 0.05 ^Bcd^	-	-
	16	2.31 ± 0.04 ^Abc^	2.12 ± 0.06 ^Bd^	-	-
	24	2.26 ± 0.03 ^Ac^	2.12 ± 0.05 ^Bd^	16.67 ± 4.71 ^Aa^	-

Note: Data are presented as mean ± SD (*n* = 3). Uppercase letters indicate intergroup differences at identical storage days, and lowercase letters indicate intra-group differences over storage time (*p* < 0.05, two-way ANOVA + Tukey’s HSD).

**Table 3:** Sensory shelf-life estimations.

Temperature (°C)	Group	CI 25% (Days)	CI 50% (Days)	PV (Days)
5	CK	4.19 ± 0.04	4.44 ± 0.06	10.96
	TG	6.11 ± 0.07	6.49 ± 0.07	14.46
−5	CK	15.73 ± 0.15	16.7 ± 0.22	15.79
	TG	21.58 ± 0.17	22.9 ± 0.15	18.83
−18	CK	102.67 ± 0.00	108.99 ± 1.38	124.27
	TG	128.83 ± 2.16	136.76 ± 2.23	224.28

## Data Availability

The original contributions presented in this study are included in the article. Further inquiries can be directed to the corresponding author.

## Appendix A

**Table A1:** Box–Behnken experimental factors and corresponding level arrangements.

Factor	Level		
	−1	0	1
sodium D-isoascorbate	0.20%	0.30%	0.40%
phospholipids	0.08%	0.10%	0.12%
sodium lactate	0.04%	0.05%	0.06%

**Table A2:** Sensory evaluation of red sour soup dumplings.

Item (Weight)	Evaluation Standard	Score
Flavor (20%)	The filling has authentic and well-balanced taste	16~20
	The filling has faint aroma without off odors	11~15
	Unpleasant off flavors are detected	0~10
Texture (20%)	The dumpling has moderate hardness and pleasant chewiness	16~20
	The dumpling is slightly soft/hard with inferior chewiness	11~15
	The dumpling is excessively soft/hard with poor chewiness	0~10
Color (20%)	The filling presents an appropriate color	16~20
	The filling color is slightly darker or lighter than the ideal state	11~15
	The filling color is overly dark or pale	0~10
Typical characteristic (20%)	Balanced sour soup aroma	16~20
	Weak sour soup flavor	11~15
	No characteristic sour soup aroma	0~10
Overall acceptability (20%)	Highly acceptable	16~20
	Moderately acceptable	11~15
	Unacceptable	0~10

**Table A3:** ANOVA table for RSM. Significance levels are indicated as follows: * for *p* < 0.05 and ** for *p* < 0.01.

Source	PV						Sensory Scores						TVC					
	*SS*	*df*	*MS*	*F*	*p*	Sig.	*SS*	*df*	*MS*	*F*	*p*	Sig.	*SS*	*df*	*MS*	*F*	*p*	Sig.
Model	31.83	9	3.54	30.27	0.0008	*	24.17	9	2.69	31.54	0.0007	*	0.0021	9	0.0002	39.92	0.0004	**
A	5.6	1	5.6	47.93	0.001	*	0.5	1	0.5	5.87	0.0599		0.0002	1	0.0002	30.84	0.0026	**
B	0.0034	1	0.0034	0.0291	0.8713		1.77	1	1.77	20.76	0.0061	*	0	1	0	2.01	0.2154	
C	0.1356	1	0.1356	1.16	0.3305		0.125	1	0.125	1.47	0.2798		0	1	0	4.65	0.0836	
AB	0.2181	1	0.2181	1.87	0.2301		0.0361	1	0.0361	0.424	0.5437		7.84 × 10^−6^	1	0.00000784	1.37	0.2948	
AC	1.12	1	1.12	9.56	0.0271	*	0.0036	1	0.0036	0.0423	0.8452		0.0001	1	0.0001	14.77	0.0121	*
BC	0.678	1	0.678	5.8	0.0609		0.0036	1	0.0036	0.0423	0.8452		0	1	0	4.02	0.1013	
A2	23.45	1	23.45	200.75	<0.0001	**	5.62	1	5.62	66.06	0.0005	*	0.0015	1	0.0015	260.11	<0.0001	**
B2	1.28	1	1.28	11	0.0211	*	17.45	1	17.45	205.01	<0.0001	**	0.0003	1	0.0003	45.92	0.0011	**
C2	0.4013	1	0.4013	3.44	0.123		0.4763	1	0.4763	5.59	0.0643		0.0001	1	0.0001	23.91	0.0045	**
Residual	0.5842	5	0.1168				0.4257	5	0.0851				0	5	0.00000573			
Lack of Fit	0.3847	3	0.1282	1.29	0.4656		0.2044	3	0.0681	0.6158	0.6673		0	3	0.000004868	0.6932	0.6361	
Pure Error	0.1995	2	0.0997				0.2213	2	0.1106				0	2	0.000007023			
Total Error	32.42	14					24.17	9	2.69	31.54	0.0007	*	0.0021	14				
R^2^	0.9820						0.9827						0.9863					
Adjusted R^2^	0.9495						0.9515						0.9616					

**Table A4:** The parameter estimations corresponding to the loglogistic distribution. The data are presented as mean ± SD.

Parameter	Estimation
β_0_	−34.01 ± 0.07
Ea_CK (kJ/mol)	82.10 ± 0.14
β_1_	9873.98 ± 16.47
β_2_	2.07 ± 0.28
β_3_	−469.50 ± 74.70
Ea_TG (kJ/mol)	78.19 ± 0.60
σ	0.05 ± 0.00
invTempK	9873.98 ± 16.47
invTempK·group	−469.50 ± 74.70

**Table A5:** Distribution comparison.

Dist	LogLik	AIC
loglogistic	−235.52	479.03
lognormal	−237.76	483.51
weibull	−247.70	503.41

**Table A6:** Kinetic model comparison.

Group	Model	AIC	AICc	BIC	R^2^	RMSE
CK	expT_piecewise_n	−62.20	−61.03	−51.99	0.952	0.53
	arrhenius_piecewise_n	−62.10	−60.92	−51.89	0.952	0.53
	arrhenius_n1	7.34	7.80	13.47	0.827	1.01
	expT_n1	9.38	9.84	15.51	0.821	1.03
TG	expT_piecewise_n	−250.80	−249.69	−240.33	0.945	0.11
	arrhenius_piecewise_n	−249.63	−248.52	−239.16	0.944	0.12
	arrhenius_n1	−172.74	−172.31	−166.46	0.783	0.26
	expT_n1	−165.85	−165.42	−159.57	0.757	0.24

**Table A7:** The parameter estimations corresponding to the expT_piecewise_n model.

Group	PV_0_	PV∞	n_chill	n_freeze	α	β	k_ref	T_ref_C	T_ref_K	Q10	E_a (KJ/mol)
CK	0.02	7.01	1.69	1.01	−3.76	0.02	0.03	5.00	278.15	1.16	8.90
TG	0.02	1.59	1.76	1.19	−3.87	0.10	0.03	5.00	278.15	2.60	58.03

## Appendix B

The results revealed that the peroxide value (PV) of meat fillings in red sour soup dumplings gradually rose with extended storage time, while the overall sensory total score exhibited a declining trend (Figure A1). After 2 days of storage, the differences in PV between the control group (CK) and other experimental groups increased significantly, and undesirable odors were detected in all samples on day 6 of storage. The treatments supplemented with sodium D-isoascorbate at concentrations above 0.30% (SE 3–5), phospholipids at 0.10% (PL 5), or sodium lactate at 0.05% (SL 5) effectively restrained PV accumulation in dumpling meat fillings throughout storage. Meanwhile, the combined treatment containing sodium D-isoascorbate, phospholipids, and sodium lactate only developed off-odors on the eighth storage day. Among single-component treatments, sodium D-isoascorbate at >0.30% (SE 3), phospholipids at 0.10% (PL 5), and sodium lactate at 0.05% (SL 5) delivered the optimal performance in preserving sensory attributes. Furthermore, no significant difference was observed in initial sensory scores (day 0) between red sour soup dumplings incorporated with the three preservatives and the untreated control, indicating that these three preservatives exerted no adverse impacts on the inherent sensory properties of red sour soup dumplings.

With the extension of storage time, the total aerobic plate count (APC) and coliform counts of meat fillings from red sour soup dumplings increased gradually (Figure A2). After 2 days of storage, the differences in microbial indicators between the untreated control group (CK) and each preservative treatment group became significantly greater. No coliforms were detected in the meat fillings of red sour soup dumplings on day 0 and day 2. Single treatments with sodium D-isoascorbate at concentrations above 0.30% (SE 3, SE 4, SE 5), phospholipids at 0.10% (PL 5), or sodium lactate at 0.05% (SL 5) exhibited the most prominent inhibitory effects on microorganisms in the meat fillings of red sour soup dumplings.

## Appendix C

### Appendix C.1. Response Surface Methodology (RSM) Optimization

The Box–Behnken design was generated and analyzed using Design-Expert software (version 13.0.1, Stat-Ease, Inc., Minneapolis, MN, USA). A total of 15 experimental runs were generated, comprising 12 factorial points and three center points. The center points were included to estimate pure error and test for lack of fit. Each experimental run was performed in randomized order to minimize systematic bias. The significance of model terms, goodness of fit, and adequacy were evaluated by analysis of variance (ANOVA) at the *p* < 0.05 significance level.

Experimental data a second-order polynomial equation to generate response surface models, describing the relationship between the independent variables and the responses. The general form of the equation is expressed as:

(A1) $$Y\;=\beta_{0}+\sum \beta_{i}X_{i}+\sum \beta_{ij}{X_{i}}^{2}+\sum \beta_{ij}X_{i}X_{j},$$

where *Y* is the predicted response; *β*_0_ is the intercept; *β*_*i*_, *β*_*i**i*_, and *β*_*i**j*_ are the linear, quadratic, and interaction coefficients, respectively; and *X*_*i*_ and *X*_*j*_ are the coded independent variables. The adequacy of the fitted models was evaluated based on the coefficient of determination (*R*^2^), adjusted *R*^2^, and the lack-of-fit test. Three-dimensional (3D) response surface plots and two-dimensional (2D) contour plots were generated to visualize the interaction effects between the variables and to determine the optimal combination of factors for minimizing PV and APC while maximizing the sensory score.

### Appendix C.2. Color Difference

Approximately 25 g (Model LQC3003Y, Jiaxing Zhengfeng Intelligent Equipment Co., Ltd., Jiaxing, China; precision ±0.001 g) of meat filling was evenly spread in a glass Petri dish of 9 cm diameter, with the thickness controlled at 0.5 cm, ensuring complete coverage of the dish bottom without air bubbles. A calibrated chroma meter (CR-400, Konica Minolta Sensing, Inc., Osaka, Japan), equipped with a D65 standard illuminant and a 2° standard observer, was employed. The instrument was calibrated against a standard white tile (Y = 93.6, x = 0.3134, y = 0.3194) prior to each measurement session. The *L** (lightness), *a** (redness/greenness), and *b** (yellowness/blueness) values were measured at 24 randomly distributed points on the sample surface, avoiding edge areas (within 1 cm from the dish periphery).

### Appendix C.3. Peroxide Value

In total, 40 g of meat filling was extracted with petroleum ether (boiling range 30–60 °C, Sinopharm Chemical Reagent Co., Ltd., Shanghai, China) in a sealed, light-protected container for 12 h. The extract was filtered through Whatman No. 1 filter paper and concentrated to dryness under reduced pressure using a rotary evaporator (Model LC-RE-2000B, Shanghai Lichen Bangxi Instrument Technology Co., Ltd., Shanghai, China; 40 °C water bath) to obtain the lipid sample. An accurately weighed portion of the extracted lipid (2.0 g) was dissolved in 25 mL of chloroform–glacial acetic acid (2:3, *v*/*v*; Tianjin Fuyu Fine Chemical Co., Ltd., Tianjin, China), followed by the addition of 1.0 mL of saturated potassium iodide solution (Sinopharm Chemical Reagent Co., Ltd., Shanghai, China). The mixture was allowed to react in the dark for 5 min, after which 100 mL of distilled water was added. The liberated iodine was titrated with 0.01 mol/L sodium thiosulfate standard solution (Tianjin Kemio Chemical Reagent Co., Ltd., Tianjin, China) using 1% (*w*/*v*) starch solution as the indicator. The results were expressed as millimoles of active oxygen per kilogram of sample (mmol/kg).

### Appendix C.4. TBARS

In total, 5 g of meat filling was mixed with 50 mL of trichloroacetic acid (TCA; Shanghai Aladdin Biochemical Technology Co., Ltd., Shanghai, China) solution (7.5%, *w*/*v*) containing 0.10% (*w*/*v*) ethylenediaminetetraacetic acid (EDTA; Shanghai Aladdin Biochemical Technology Co., Ltd., Shanghai, China) and shaken at 50 °C for 30 min in a constant temperature (Model 2D-85, Shanghai Lichen-BX Instrument Technology Co., Ltd., Shanghai, China). The mixture was filtered through Whatman No. 1 filter paper, and 5.0 mL of the filtrate was mixed with 5.0 mL of 0.02 mol/L thiobarbituric acid (TBA) aqueous solution. The mixture was heated in a 90 °C water bath for 30 min, cooled to room temperature, and the absorbance was measured at 532 nm using a UV–Vis spectrophotometer (Model UV-2550, Shimadzu Corporation, Kyoto, Japan). The results were expressed as milligrams of malondialdehyde (MDA) equivalents per kilogram of sample (mg MDA/kg).

### Appendix C.5. TVB-N

In total, 10 g of meat filling was homogenized with 75 mL of 0.85% NaCl and allowed to stand for 30 min with intermittent shaking. Following the addition of 1.0 g of magnesium oxide, the mixture was immediately connected to the distillation apparatus. The distillation parameters were set as follows: alkali and water addition volumes 0 mL, boric acid receiving solution (Chengdu Dushanjin Chemical Reagent Co., Ltd., Chengdu, China) 30 mL, and distillation time 180 s. Ten drops of methyl red–bromocresol green mixed indicator (ethanolic solution; Jinshi Yongda Chemical Reagent Co., Ltd., Tianjin, China) were added to the receiving flask, and the distillate was titrated with 0.10 mol/L hydrochloric acid standard solution (Sinopharm Chemical Reagent Co., Ltd., Shanghai, China) to a gray-blue endpoint. The results were expressed as mg N per 100 g of sample (mg N/100 g).

### Appendix C.6. APC and Coliform Counts

In total, 5 g (Model LQC3003Y, Jiaxing Zhengfeng Intelligent Equipment Co., Ltd., Jiaxing, China; precision ±0.001 g) of meat filling was aseptically transferred to a sterile stomacher bag containing 45 mL of sterile physiological saline (0.85%, *w*/*v* NaCl) and homogenized using a stomacher blender for 120 s. The resulting suspension was subjected to tenfold serial dilutions.

Aerobic plate count (APC): One milliliter of the diluted sample was transferred to a sterile Petri dish and overlaid with 15–20 mL of molten plate count agar (PCA; Shanghai Bowei Biotechnology Co., Ltd., Shanghai, China). After solidification, the plates were incubated at 36 ± 1 °C in an electrothermal incubator (Model DH5000BII, Tianjin Taisite Instrument Co., Ltd., Tianjin China) for 48 ± 2 h, and colonies were enumerated and expressed as colony-forming units per gram (CFU/g).

Coliform count: The VRBA pour-plate method was employed using violet, red bile agar (VRBA; Shanghai Bowei Biotechnology Co., Ltd., Shanghai China), incubated at 36 ± 1 °C for 24 ± 2 h. Typical colonies were picked and inoculated into brilliant green lactose bile broth (BGLB; Shanghai Bowei Biotechnology Co., Ltd., Shanghai China), incubated at 36 ± 1 °C for 48 ± 2 h. The coliform count was calculated as the most probable number per gram (MPN/g) based on the number of gas-positive tubes. Plates yielding 30–300 colonies were selected for counting. The results were expressed as logarithm of CFU/g (log CFU/g) or logarithm of MPN/g (log MPN/g). All determinations were performed in triplicate.

### Appendix C.7. Sensory Evaluation

The trained sensory panel comprised 50 assessors (25 females and 25 males, aged 18–25 years) selected, all of whom had completed a professional training program in meat product sensory evaluation prior to the study. Crucially, the same panel of judges evaluated the samples throughout the entire storage period to ensure consistency in the assessment. Panelists were trained in attribute recognition, intensity scaling, and scoring consistency over a 3-day training period using commercial dumpling products spanning the full quality spectrum as reference anchors. All assessors provided written informed consent prior to participation. Each attribute was evaluated on a 20-point line scale (0 = intensity absent/very poor; 20 = intensity very strong/excellent). The overall acceptability rating was recorded as a binary acceptable/unacceptable judgment for subsequent survival analysis modeling as shown in Table A2.

Frozen dumplings were cooked in boiling water (100 °C) for 15 min, removed, and placed in coded disposable white plastic trays. Samples were allowed to cool down to 50 ± 2 °C (measured using a digital probe thermometer) before serving. The evaluation was under natural daylight-equivalent fluorescent lighting (color temperature 6500 K) at a controlled ambient temperature of 20 ± 1 °C and 60–70% relative humidity. The samples were randomly three-digit coded and presented to panelists in a balanced, randomized order. Room-temperature water was provided as palate cleansers between samples. Each panelist evaluated one sample per session, with a minimum 30 min inter-sample interval.

### Appendix C.8. Textural Properties

A texture analyzer (EZ-Test, Shimadzu Corp Ltd., Kyoto, Japan ) was used for texture profile analysis (TPA); the instrument was equipped with a P/5 probe (Stable Micro Systems) and operated at a pre-test speed of 0.5 mm/min, a deformation distance of 10 mm, a hold time of 30 s, and a post-test speed of 500 mm/s, to determine hardness and springiness. For sheer force determination, the texture analyzer was set to shear mode using a shear probe (Stable Micro Systems) at a pre-test speed of 30 mm/min, a shear distance of 40 mm, and a post-test speed of 500 mm/s, to determine tenderness and chewiness.

### Appendix C.9. Sensory Shelf Life

Sensory shelf life was modeled using parametric accelerated failure time (AFT) models based on interval-censored rejection data. Model parameterization was performed using a three-point temperature gradient dataset, comprising accelerated shelf-life testing (ASLT) data acquired at 5 °C and −5 °C, supplemented by an additional long-term storage experiment conducted at −18 °C with intensive sampling at 0, 30, 50, 65, 80, 95, 110, and 125 d. In consumer rejection tests, panelists periodically evaluated product acceptability during storage and indicated whether the sample was acceptable or unacceptable. Because the exact rejection time is unknown but occurs between two observation times, the rejection data was treated as interval-censored. For each consumer under each storage temperature and treatment condition, the rejection interval was defined as the time between the last accepted observation and the first rejected observation. If a consumer never rejected the product during the test period, the observation was treated as right-censored. Conversely, if rejection occurred at the first observation point, the data would be treated as left-censored. Parametric AFT models were fitted to the interval-censored data using three candidate distributions: Weibull, lognormal, and loglogistic.

The interaction term between inverse temperature and treatment was included to evaluate the temperature sensitivity of sensory deterioration in the TG. Model parameters were estimated by maximum likelihood. To account for repeated observations from the same consumer at different temperatures, cluster-robust standard errors were calculated using temperature–consumer combinations as clustering units.

Sensory shelf life was defined as the storage time corresponding to a 50% rejection probability, which was obtained from the fitted AFT model. Predicted rejection curves and shelf-life estimates at different storage temperatures were derived from the final model. All analyses were conducted using Python 3.13 (Python Software Foundation, https://www.python.org/) with custom scripts implementing maximum likelihood estimation for interval-censored AFT models.

### Appendix C.10. Peroxide Value Shelf Life

To account for potential differences between chilling and freezing conditions, a piecewise formulation was applied in which the shape parameter n varied between two temperature regions: $n=n_{chill}$ for T≥−10 °C and $n=n_{freeze}$ for T<−10 °C. Four candidate models were evaluated, including expT_piecewise_n, expT_n1, arrhenius_piecewise_n, and arrhenius_n1. Model parameters were estimated to use nonlinear least-squares regression with a multi-start fitting strategy to avoid convergence to local minima, and a soft-L1 robust loss function was applied to reduce the influence of outliers. Model performance was evaluated using the coefficient of determination (R^2^), root mean square error (RMSE), Akaike information criterion (AIC), corrected AIC (AICc), and Bayesian information criterion (BIC). The model with the lowest AIC value was selected as the optimal model for describing PV kinetics. All analyses were performed in Python using the SciPy optimization library.

## References

[1] Li D., Duan F., Tian Q., Zhong D., Wang X., Jia L. (2021). Physiochemical, microbiological and flavor characteristics of traditional Chinese fermented food Kaili Red Sour Soup. LWT.

[2] Wang C., Zhang Q., He L., Li C. (2020). Determination of the microbial communities of Guizhou Suantang, a traditional Chinese fermented sour soup, and correlation between the identified microorganisms and volatile compounds. Food Res. Int..

[3] Lin L.-J., Zeng J., Tian Q.-M., Ding X.-Q., Zhang X.-Y., Gao X.-Y. (2022). Effect of the bacterial community on the volatile flavour profile of a Chinese fermented condiment–Red sour soup–During fermentation. Food Res. Int..

[4] Zhou X., Liu Z., Xie L., Li L., Zhou W., Zhao L. (2022). The correlation mechanism between dominant bacteria and primary metabolites during fermentation of red sour soup. Foods.

[5] Li C., Zhang Q., Wang C., He L., Tao H., Zeng X., Dai Y. (2022). Effect of starters on quality characteristics of Hongsuantang, a Chinese traditional sour soup. Fermentation.

[6] Bao X., Zhang S., Zhang X., Jiang Y., Liu Z., Hu X., Yi J. (2022). Effects of pasteurization technologies and storage conditions on the flavor changes in acidified chili pepper. Curr. Res. Food Sci..

[7] Xia Y., Eerdun B., Wang J., Li Y., Shuang Q., Chen Y. (2023). Variation and Correlation Analysis of Flavour and Bacterial Diversity of Low-Salt Hotpot Sauce during Storage. Foods.

[8] Li F., Zhong Q., Kong B., Wang B., Pan N., Xia X. (2020). Deterioration in quality of quick-frozen pork patties induced by changes in protein structure and lipid and protein oxidation during frozen storage. Food Res. Int..

[9] Huang L., Kong B., Zhao J., Liu Q., Diao X. (2014). Contributions of fat content and oxidation to the changes in physicochemical and sensory attributes of pork dumpling filler during frozen storage. J. Agric. Food Chem..

[10] Huang L., Xiong Y.L., Kong B., Huang X., Li J. (2013). Influence of storage temperature and duration on lipid and protein oxidation and flavour changes in frozen pork dumpling filler. Meat Sci..

[11] Zhang T., Dai S., Niu B., Shen C., Chen H., Liu R., Chen H., Wu W., Gao H. (2025). Investigating the mechanism behind warmed-over flavor in prepared pork products during their shelf life using GC-IMS. Food Chem. X.

[12] Huang L., Liu Q., Xia X., Kong B., Xiong Y.L. (2015). Oxidative changes and weakened gelling ability of salt-extracted protein are responsible for textural losses in dumpling meat fillings during frozen storage. Food Chem..

[13] Sepe H.A., Faustman C., Lee S., Tang J., Suman S.P., Venkitanarayanan K.S. (2005). Effects of reducing agents on premature browning in ground beef. Food Chem..

[14] Tenderis B., Kılıç B., Yalçın H., Şimşek A. (2020). Impact of sodium lactate, encapsulated or unencapsulated polyphosphates and their combinations on Salmonella Typhimurium, Escherichia coli O157:H7 and Staphylococcus aureus growth in cooked ground beef. Int. J. Food Microbiol..

[15] Liu J., Huang M., Mao Y., Zhang Y., Zhu L., Zuo H. (2026). Phospholipid-myosin non-covalent binding in emulsified meat systems: Interfacial behavior and conformational changes. Food Chem..

[16] do Vale R.B., Barros A.C., Maciel I.d.J., Melo S.K.d.S., da Silva T.R.R., da Silva J.J., da Silva J.A., Lima J.d.S., de Alencar M.G., da Costa M.M. (2026). The Effects of Substituting BHT with a Microencapsulated Basil (Ocimum campechianum Mill.) Extract on Refrigerated Beef Burger Preservation. Foods.

[17] Zhang Z., Du L., Jiang P., Chen D., Liu M., Guo F., Zhang C. (2026). High-voltage electrostatic field thawing stabilizes water immobilization and preserves ventral-skin golden appearance in frozen large yellow croaker (*Larimichthys crocea*). Food Chem. X.

[18] Sajadi M.S., Sharifi Soltani M., Ariaii P., Jafarian S. (2024). Evaluation of the effect of a composite coating of chitosan-cress seed gum, along with hydrolyzed protein from sesame meal (in free and encapsulated forms), to increase the shelf life of ostrich fillets. J. Food Meas. Charact..

[19] Liu F., Wang C., Li C., He L., Wang X., Zeng X., Dai Y. (2022). Effects of Process Parameters on the Quality of Suantang Beef. Foods.

[20] Pan Z., Bai Y., Xu L., Zhang Y., Lei M., Huang Z. (2023). The effect of freeze–thaw cycles on the microscopic properties of dumpling wrappers. Foods.

[21] Hough G., Garitta L., Gómez G. (2006). Sensory shelf-life predictions by survival analysis accelerated storage models. Food Qual. Prefer..

[22] Peleg M., Corradini M.G., Normand M.D. (2004). Kinetic Models of Complex Biochemical Reactions and Biological Processes. Chem. Ing. Tech..

[23] Richards M.P. (2013). Redox reactions of myoglobin. Antioxid. Redox Signal..

[24] Suman S.P., Joseph P. (2013). Myoglobin chemistry and meat color. Annu. Rev. Food Sci. Technol..

[25] Baron C.P., Andersen H.J. (2002). Myoglobin-Induced Lipid Oxidation. A Review. J. Agric. Food Chem..

[26] Faustman C., Sun Q., Mancini R., Suman S.P. (2010). Myoglobin and lipid oxidation interactions: Mechanistic bases and control. Meat Sci..

[27] Lee S., Decker E., Faustman C., Mancini R. (2005). The effects of antioxidant combinations on color and lipid oxidation in n− 3 oil fortified ground beef patties. Meat Sci..

[28] Kim Y.H., Hunt M.C., Mancini R.A., Seyfert M., Loughin T.M., Kropf D.H., Smith J.S. (2006). Mechanism for lactate-color stabilization in injection-enhanced beef. J. Agric. Food Chem..

[29] McClure B.N., Sebranek J.G., Kim Y.H., Sullivan G.A. (2011). The effects of lactate on nitrosylmyoglobin formation from nitrite and metmyoglobin in a cured meat system. Food Chem..

[30] Kröger-Ohlsen M., Skibsted L.H. (1997). Kinetics and Mechanism of Reduction of Ferrylmyoglobin by Ascorbate and d-Isoascorbate. J. Agric. Food Chem..

[31] Lee K., Shimaoka J. (1984). Forms of iron in meats cured with nitrite and erythorbate. J. Food Sci..

[32] Fox J.B., Ackerman S.A. (1968). Formation of Nitric Oxide Myoglobin: Mechanisms of the Reaction with Various Reductants. J. Food Sci..

[33] Reith J., Szakaly M. (1967). Formation and stability of nitric oxide myoglobin I. Studies with model systems. J. Food Sci..

[34] Shang X., Zhou Z., Jiang S., Guo H., Lu Y. (2020). Interrelationship between myoglobin oxidation and lipid oxidation during the processing of Cantonese sausage with d-sodium erythorbate. J. Sci. Food Agric..

[35] Wang Z., He Z., Gan X., Li H. (2018). Interrelationship among ferrous myoglobin, lipid and protein oxidations in rabbit meat during refrigerated and superchilled storage. Meat Sci..

[36] Domínguez R., Pateiro M., Munekata P.E.S., Zhang W., Garcia-Oliveira P., Carpena M., Prieto M.A., Bohrer B., Lorenzo J.M. (2022). Protein Oxidation in Muscle Foods: A Comprehensive Review. Antioxidants.

[37] Lee S., Jo K., Jeong H.G., Choi Y.-S., Kyoung H., Jung S. (2024). Freezing-induced denaturation of myofibrillar proteins in frozen meat. Crit. Rev. Food Sci. Nutr..

[38] Han J., Wang Y., Wang Y., Hao S., Zhang K., Tian J., Jin Y. (2024). Effect of changes in the structure of myoglobin on the color of meat products. Food Mater. Res..

[39] Wu C., Valenta J., Hamilton E., Modrow K., Osburn W. (2019). The Optimization of the Concentrations of Sodium Lactate (Nal), Sodium Erythorbate (Nae), and Sodium Bicarbonate (Nab) Applied to Beef Trimmings for Ground Beef Production. Meat Muscle Biol..

[40] Dai H., Sun Y., Xia W., Ma L., Li L., Wang Q., Zhang Y. (2021). Effect of phospholipids on the physicochemical properties of myofibrillar proteins solution mediated by NaCl concentration. LWT.

[41] Yang B., Lan M., Zhong R., Shi F., Liang P. (2024). Insight into the effects of large yellow croaker roe (*Larimichthys crocea*) phospholipids on the conformational and functional properties of pork myofibrillar protein. Food Chem..

[42] Yang B., Lan M., Liu X., Liu J., Xiong W., Zhang M., Liang P. (2025). Integrated experiments and molecular dynamics simulation to decipher the interaction of marine phospholipids with myosin: Focus on binding mechanisms, conformation, and functional properties. Food Res. Int..

[43] Xia X., Kong B., Xiong Y., Ren Y. (2010). Decreased gelling and emulsifying properties of myofibrillar protein from repeatedly frozen-thawed porcine longissimus muscle are due to protein denaturation and susceptibility to aggregation. Meat Sci..

[44] Xia W., Ma L., Chen X., Li X., Zhang Y. (2018). Physicochemical and structural properties of composite gels prepared with myofibrillar protein and lecithin at various ionic strengths. Food Hydrocoll..

[45] Zhou X., Lin H., Zhu S., Xu X., Lyu F., Ding Y. (2020). Textural, rheological and chemical properties of surimi nutritionally-enhanced with lecithin. LWT.

[46] Ding Y., Ding Y., Qi Y., Shen Z., Xu Y., Yu D., Xia W. (2026). Effects of different lipids on the freeze-thaw stability and subsequent gelling properties of emulsified myofibrillar protein sols: Perspective on interfacial behavior. Food Chem. X.

[47] Kim H., Hong G.-P. (2022). Comparison of Superchilling and Supercooling on Extending the Fresh Quality of Beef Loin. Foods.

[48] Yi J., Chen F., Li H., Yu Q., Sun C., Wen R. (2025). Oxidative–textural coupling mechanisms in chicken breast stored at low-temperature gradients: Super-chilling as a quality preserving strategy. Food Res. Int..

[49] Limbo S., Torri L., Sinelli N., Franzetti L., Casiraghi E. (2010). Evaluation and predictive modeling of shelf life of minced beef stored in high-oxygen modified atmosphere packaging at different temperatures. Meat Sci..

[50] Hough G., Langohr K., Gómez G., Curia A. (2003). Survival Analysis Applied to Sensory Shelf Life of Foods. J. Food Sci..

[51] Garitta L., Langohr K., Elizagoyen E., Gugole Ottaviano F., Gómez G., Hough G. (2018). Survival analysis model to estimate sensory shelf life with temperature and illumination as accelerating factors. Food Qual. Prefer..

[52] Lu X., Zhang Y., Zhu L., Luo X., Hopkins D.L. (2019). Effect of superchilled storage on shelf life and quality characteristics of M. longissimus lumborum from Chinese Yellow cattle. Meat Sci..

[53] Lu X., Zhang Y., Xu B., Zhu L., Luo X. (2020). Protein degradation and structure changes of beef muscle during superchilled storage. Meat Sci..

[54] Wang Z., He Z., Zhang D., Li H., Wang Z. (2020). Using oxidation kinetic models to predict the quality indices of rabbit meat under different storage temperatures. Meat Sci..

[55] Wang B., Wang S., Xu Y., Jiang Q., Xia W. (2024). Colour stability improving of yellowfin tuna slices by compound antioxidant against oxidation of myoglobin. Int. J. Food Sci. Technol..

[56] Amaral A.M.P.D., Amorim E.M., Carmo E.G.S.D., Santos J.V.P.d., Canova M.T., Dalan P.A., Bettanin L., Robazza W.D.S., Moroni L.S., Sehn G.A.R. (2025). Different commercial technological ingredients: Impact on the shelf life of refrigerated fresh sausages. Ciênc. Anim. Bras..

[57] Jiang L.-S., Li Y.-C., Zheng F.-X., Zhang M.-J., Zheng W.-X., Liu D.-Y., Meng F.-B. (2024). Application of Lactiplantibacillus plantarum hydrogel coating in combination with ice temperature for the preservation of fresh yak meat. Food Chem. X.

[58] You L., Qi W., Zhang Z., Sun Y., Wang J., Jiang Y., Liu B., Hu N., Song J. (2026). Effects of Acetic Acid Pretreatment Combined With Composite Preservation Methods on Protein and Quality Characteristics of Chilled Venison. J. Food Process. Preserv..

